# DNA Methylation Variation in Blood Cells may Impact Platelet Function

**DOI:** 10.1055/a-2883-9068

**Published:** 2026-06-19

**Authors:** Jillian Teichman, Ming-Huei Chen, Florian Thibord, Melissa V. Chan, Bongani B. Nkambule, Amber R. Lachapelle, Jiantao Ma, Roby Joehanes, Chunyu Liu, Angel M. Aponte, Sumith R. Panicker, Yogendra Kanthi, Daniel Levy, Andrew D. Johnson

**Affiliations:** 1 The Framingham Heart StudyNational Heart, Lung, and Blood Institute, National Institutes of HealthFraminghamMassachusettsUnited States; 2 Population Sciences BranchDivision of Intramural ResearchNational Heart, Lung, and Blood Institute, National Institutes of HealthFraminghamMassachusettsUnited States; 3 Bordeaux Population Health Research CenterUniversity of BordeauxTalenceNouvelle-AquitaineFrance; 4 School of Laboratory Medicine and Medical SciencesCollege of Health Sciences, University of KwaZulu-NatalDurbanKwaZulu-NatalSouth Africa; 5 Friedman School of Nutrition Science and PolicyTufts UniversityBostonMassachusettsUnited States; 6 Department of BiostatisticsBoston UniversityBostonMassachusettsUnited States; 7 Population Sciences BranchDivision of Intramural ResearchNational Heart, Lung, and Blood Institute, National Institutes of HealthBethesdaMarylandUnited States; 8 Section of Vascular Thrombosis and InflammationDivision of Intramural ResearchNational Heart, Lung, and Blood Institute, National Institutes of HealthBethesdaMarylandUnited States

**Keywords:** megakaryocytes, DNA methylation, platelet reactivity, epigenome, EWAS

## Abstract

DNA methylation modifies nucleotides, regulating gene expression without sequence change. The effects of DNA methylation within blood cell lineages on the development and function of endomitotic high DNA copy megakaryocytes (MKs) to anucleate platelets remain poorly understood. We sought to characterize this potential relationship by investigating associations between platelet function and methylation in circulating nucleated blood cells. Data were measured in the Framingham Heart Study Third Generation cohort ( *n* = 1,314). Five bioassays assessed platelet function in response to up to seven agonists in whole blood and platelet-rich plasma, and the Illumina 450K array was used to conduct an epigenome study of blood DNA methylation. In adjusted statistical association models, we found 46 significant associations (false discovery rate-adjusted *p* < 0.05) across 36 genomic DNA methylation sites, including cg24267699 in a putative regulatory site −742/−743 bases upstream from the *ABO* transcription start site associated with ristocetin platelet agglutination ( *β* = 0.21, standard error = 0.03, *p* < 1.04E-11). The 36 sites collectively reside within genes acting as transcription factors, genes implicated in granule release and exocytosis, cytoskeletal functions, mitochondrial function, and platelet function. The set of associated cytosine-phosphate-guanines was enriched in MKs for markers of regulatory activity, including DNase-I hypersensitivity sites and histone activity. Overall, we report in the first such epigenome scan that blood cell DNA methylation appears to be significantly associated with several platelet reactivity traits, and may drive the regulation of key genes involved in those processes presumably at the upstream level of hematopoietic stem cells or megaerythroid lineage cells.

## Introduction

DNA methylation is the process of adding methyl groups to nucleotides, ultimately enabling the postsynthesis modification of DNA without sequence change. This is a dynamic process—DNA methylation profiles are known to change with age, smoking exposure, and cancer status, among many other environmental factors. 1 2 3 4 Differential DNA methylation is known to alter gene expression in a tissue-specific fashion, 5 with a multitude of implications for mammalian developmental maturation and disease etiology. 1 6 These modifications typically occur on cytosine nucleotides directly followed by a guanine nucleotide, and are also referred to as cytosine-phosphate-guanine (CpG) sites. In recent years, epigenome-wide association studies (EWASs) have successfully identified associations with numerous phenotypes and DNA methylation patterns, specifically in leukocytes; in addition to identifying disease-risk variants and biomarkers of drug responses. 7 8

However, the role of leukocyte DNA methylation in influencing the biological processes of platelets is poorly understood. Prior work has identified specific loci, such as CpGs within and around *F2RL3* (encoding the human thrombin receptor protein PAR4) and *PEAR1* , which are known to be associated with cardiovascular outcomes and platelet function. 9 10 Another study found that circulating fibrinogen levels and DNA methylation in peripheral blood leukocytes are significantly associated at 83 CpGs, highlighting a potential inflammation-driven mechanism of fibrinogen regulation. 11 Further still, a few studies have examined platelet mitochondrial DNA methylation patterns and found associations with altered methylation and various phenotypes, such as electrocardiogram abnormalities and cardiovascular outcomes in individuals with obesity. 12 13 Simultaneously, work to characterize altered methylation profiles during megakaryopoiesis and maturation is ongoing. 14 15 16 Currently, there is a paucity of holistic, genome-wide population-level research on the role of methylation in nucleated blood cells, directly or indirectly through lineage precursors, on downstream platelet function. As platelets are essential for normal inflammation responses, hemostasis, and thrombosis, this void presents an area of interest in understanding platelet function outcomes.

In the interest of elucidating these potential relationships, we aimed to identify associations between DNA methylation in circulating white blood cells (WBCs) and platelet functional traits. We utilized five assays to evaluate platelet reactivity, and measured DNA methylation via the Illumina 450K platform in Framingham Heart Study (FHS) Third Generation Cohort participants. We integrated significant results with regulatory markers in megakaryocyte (MK) samples from the Blueprint project.

## Methods

### Framingham Heart Study Sample

The FHS is a community-based longitudinal cohort study following three generations of participants, beginning in 1948. Cross-sectional data were collected from the third exam of the Third Generation cohort (2016–2019), consisting of a total of 3,089 individuals. Beginning recruitment in 2002, the Third Generation cohort is composed of adults who have at least one parent in the FHS Offspring cohort. Recruitment and epidemiological details have been previously reported. 17 From this pool, a total of 1,314 Third Generation participants had platelet functional trait data and methylation data.

### Measurement of DNA Methylation

In a prior study, 18 19 DNA methylation levels were measured in multiple cell types isolated from whole blood (WB) buffy coat samples, prepared using previously described protocols. We utilized methylation data measured from the Third Generation cohort during the second exam (2008–2011). 18 Bisulfite conversion was used to prepare samples for whole-genome amplification, fragmentation, array hybridization, and single-base pair (bp) extension. The Illumina (San Diego, California, United States) Infinium Human Methylation-450 Beadchip (450K array) was then used to measure DNA methylation in 1,314 participants, taking place over the course of three batches. No more than 5% of CpGs were missing methylation level measurements in all participants. Measured as a range of 0 to 1, DNA methylation β values were calculated as the ratio of mean methylated probe signal intensity to the sum of methylated and unmethylated probe signal intensities. 19 Normalization of methylation β values was performed via the DASEN method. 20

### Blood Collection and Platelet Assays

During the third exam (2016–2019), participants fasted overnight, and blood was drawn the subsequent morning in a supine position into glass sodium citrate (3.2%; BD) and hirudin-anticoagulated (Diapharma, Roche Diagnostics, Indianapolis, Indiana, United States) vacutainers. Blood samples were processed at room temperature in accordance with the International Society on Thrombosis and Hemostasis Guidelines. 21 Platelet reactivity was assessed via five bioassays, with detailed platelet assay parameters previously described. 22

From hirudin-anticoagulated WB, platelet function tests were conducted using the Multiplate (MP) system (Roche Diagnostics, Indianapolis, Indiana, United States) and T-TAS automated microchip flow chamber (PL-chip; Zacros, Tokyo, Japan). Isolated platelet-rich plasma (PRP) was utilized for light transmission aggregometry (LTA) in an 8-channel aggregometer (Bio/Data, Horsham, Pennsylvania, United States), and Optimul 96-well aggregometry. 23 An Accuri C6 (BD Biosciences, Franklin Lakes, New Jersey, United States) assessed flow cytometry (FC) responses in hirudin-anticoagulated WB and PRP. Tested platelet agonists across all platforms included various concentrations of adenosine diphosphate (ADP), arachidonic acid (AA), collagen, epinephrine, ristocetin, thrombin receptor activator peptide 6 (TRAP-6), and U46619. Save for AA (Roche Diagnostics, Indianapolis, Indiana, United States) used in Multiplate, and agonists in a limited batch of Optimul plates, all agonists were obtained from Bio/Data Corporation (Horsham, Pennsylvania, United States). 22 Platelet reactivity positive β interpretations and other assay information can be found in ( Supplementary Table S1 ).

**Table 1:** Demographic and health characteristics of study sample at Exam 3

*n* -Participants = 1,314	Mean (standard deviation)/ *n* (%)
Age (y)	53.22 (7.82)
Sex (female)	690 (52.5)
Current smoker (as of 3rd exam; *n* , %)	86 (6.55)
Body mass index (BMI, kg/m ^2^ )	28.31 (5.71)
Diabetes ( *n* , %)	93 (7.08)
Systolic blood pressure (mm Hg)	117.96 (13.61)
Diastolic blood pressure (mm Hg)	75.64 (8.63)
Hypertension ( *n* , %)	650 (49.47)
Fasting glucose (mg/dL)	99.05 (19.49)
Triglycerides (mg/dL)	110.10 (70.95)
High density lipoprotein (mg/dL)	59.59 (19.03)
Low density lipoprotein (mg/dL)	107.94 (30.40)
Alcohol (drinks/wk)	4.33 (3.81)

Platelet counts (PLT) were estimated by direct volume counting using FC of PRP or WB during the third exam. Complete blood cell counts were collected at the second exam with a cell counter (Beckman-Coulter, Brea, California, United States) to measure PLT and mean platelet volume. 24

### Flow Cytometry Quality Control

To minimize the impact of abnormal events during acquisition and those affected by technical artifacts, the Flow AI package 25 (version 1.12.7) on FlowJo software (version 10.7.1, BD Biosciences, Franklin Lakes, New Jersey, United States) was used on all compensated FC files. FlowAI screens for and selects bad-quality cell events based on flow rate abnormalities, parameter instabilities, and omission of fluorescence signals outside of the dynamic range of each fluorescence channel.

### Statistical Analysis

Exclusion criteria included lack of DNA methylation measurements and missing covariates; in addition to participants whose blood samples were unable to be collected, were hemolyzed, or were lipidemic. An inverse normal transformation was applied to platelet functional data, and DNA methylation β values were standardized. A linear mixed effects model implemented in the *lmekin* function of the *coxme* R package, 26 which accounted for familial correlation, was used to test associations between DNA methylation and platelet functional traits. The model adjusted for age, sex, body mass index (BMI), aspirin use, smoking status, Optimul plate batch effects (in analysis of Optimul traits), and six estimated differential leukocyte counts (CD8+ T-cells, CD4+ T-cells, NK cells, B cells, monocytes, and granulocytes) using the Houseman method. 27 Multiple test correction was performed to control the false discovery rate (FDR) with the Benjamini and Hochberg 1995 method 28 and FDR ≤ 0.05 for each trait was reported. To ensure a lack of effects derived from medication usage, we also tested the same model with adjustments for P2Y12 inhibitors, and other antiplatelet, antidepressant, and anticoagulant drugs.

Additionally, we assessed associations between von Willebrand factor (VWF) plasma levels measured in the third exam and leukocyte DNA methylation. VWF levels were assayed on platelet-poor plasma, with samples stored at −80°C until assayed. All samples were assayed on their first freeze-thaw. VWF was measured using Abcam (Cambridge, United Kingdom) Simple-Step ELISA kits (ab223864) with a 1:6,000 dilution in three steps and adjusted to a standard curve. 29 To identify associations between VWF and leukocyte DNA methylation, we again implemented a linear mixed effects model using the *lmekin* function of the *coxme* R package. 26 The model accounted for familial correlation and adjusted for age, sex, BMI, smoking status, and six estimated differential leukocyte counts (listed above) using the Houseman method. Multiple test correction was performed to control the FDR with the Benjamini and Hochberg 1995 method, 28 and FDR ≤ 0.05 for the VWF levels association was reported.

### Analysis of Megakaryocyte Regulatory Feature Overlaps in Blueprint

To assess potential overlaps of our significant associations in gene regulatory samples in MKs, we utilized publicly available epigenetic data from Blueprint. 30 MK methylation calls from two samples (S004AV and S004BT) were compiled, with each call corresponding to a region denoted by start and end coordinates that contain multiple CpGs. Regions were globally defined as either hypo or hypermethylated in MKs, with an average methylation level given between 0 and 1, where < 0.5 corresponds to hypomethylation. The CpG sites significantly associated with platelet traits were aligned with the methylation calls from samples S004AV and S004BT. Similarly, MK DNase hypersensitivity calls (an indicator of open chromatin and regulatory activity) from two samples (C0006NS, which had two different experiments, and S004BT) and MK histone ChIP-seq calls from three samples (S004AV, S004BT, and S00VHK) were compiled and aligned against significant CpG sites. For the ChIP-seq data, six histone modifications were assessed, though sample S004AV measures are absent for H3K9me3, H3K27ac, and H3K27me3 modifications.

### Identification of Significant Overlapping DNA Methylation and Platelet RNA Level Associations

To identify associations between leukocyte DNA methylation and platelet RNA levels, a linear mixed model was implemented in the *lmekin* function of the *coxme* R package 26 that additionally adjusted for family relatedness. Data from 206 individuals in the Generation 3 cohort with both DNA methylation (measured in Exam 2) and RNA-seq measurements (measured in Exam 3) were used for analysis. Residuals were inverse normalized from regression, and the model was adjusted for age, sex, BMI, and 20 RNA sequencing principal components. From these results, we identified any overlaps with significant RNA-to-platelet function associations that were annotated to the same gene as our significant CpG-to-platelet function associations. The platelet RNA-seq data was derived from RNA isolated from PRP with miRVana kits (ThermoFisher, Waltham, Massachusetts, United States). 31 Illumina (San Diego, California, United States) TruSeq stranded kits were used with paired-end 100 cycle sequencing on an Illumina NovaSeq-6000 instrument. The *nfcor/rnaseq* pipeline (version 2.3) was run with STAR alignment and RSEM quantification against Gencode v.30. After removing samples with evidence for white cell contamination (based on CD45/CD41B ratio > 1.0), and matching against those with available DNA methylation measurements, there were *n* = 206 samples remaining.

## Results

### Participant Sample

Our study analyzed data from 1,314 Third Generation participants with both DNA methylation at Exam 2 and platelet assays at Exam 3. Platelet assay sample size variation can be attributed to differences in availability of blood collection throughout Exam 3; in addition to other factors such as blood sample quality and assay reagent availability over the multi-year exam period as previously described. 22 The main limiting factor was availability of participants with DNA methylation measurements. The final study sample had a mean age of 53.2 years (standard deviation: ± 7.82), 52.5% of participants were women, and the mean BMI was 28.3 kg/m ^2^ (standard deviation: ± 5.71). Current smokers represented 6.5% of the samples at the time of the third exam, and 7.1% of the samples were from people with diabetes. Additional study sample details are found in Table 1 . Major medications used in both exams are described in Table 2 .

**Table 2:** Medications in exams 2 and 3

Medication list	Exam cycle	
	Exam 2 (2008–2011)/ *n* (%; DNA methylation exam)	Exam 3 (2016–2019)/ *n* (%; platelet reactivity exam)
Antihypertensive drugs	219 (16.67)	326 (24.81)
Aspirin and/or other NSAID	NA a	251 (19.10)
Antidepressant drugs	187 (14.23)	238 (18.11)
–Serotonin-affecting	173 (13.17)	217 (16.51)
Statins (antilipid)	155 (11.80)	260 (19.79)
Antidiabetes drugs	38 (2.89)	70 (5.33)
Anticoagulants	4 (0.30)	16 (1.22)
–Warfarin	4 (0.30)	6 (0.46)
–Rivaroxaban	0	4 (0.30)
–Apixaban	0	4 (0.30)
–Dabigatran	0	2 (0.15)
P2Y12 Inhibitors	3 (0.23)	10 (0.76)
–Clopidogrel	3 (0.23)	9 (0.68)
–Prasugel	0	1 (0.08)
Other antiplatelet drugs	9 (0.68)	14 (1.07) b
–Sildenafil	5 (0.38)	8 (0.61)
–Tadalafil	3 (0.23)	7 (0.53)
–Vardenafil	1 (0.08)	0

a Platelet assays with arachidonic acid were not collected at Exam 2, so recent aspirin/NSAID use could not be determined with sensitivity at Exam 2.

b One individual reports use of both sildenafil and tadalafil.

### DNA Methylation Associations with Platelet Reactivity Traits

**Fig. 1:**
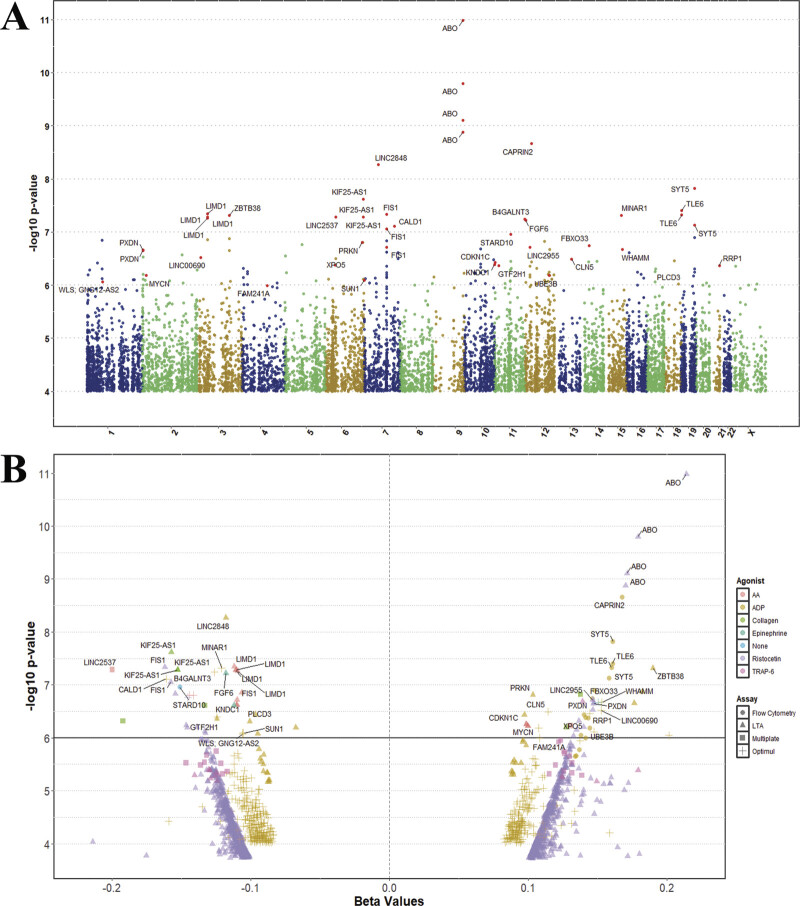
( **A** ) Manhattan plot of all associations with an FDR ≤ 0.1. Position in the hg19/GRCh37 genome is indicated in chromosomal order along the *x* -axis, with each chromosome demarcated by color. Original *p-* values were used for the plot; thus points that did not survive multiple testing are included as well. Genes that survived multiple testing and are significant are highlighted in red, and significant hits annotated to known genes are indicated with labels. ( **B** ) Volcano plot of all betas that met an FDR ≤ 0.1. The color of each point indicates the measured agonist, while the shape of points indicates the measured assay. Significant hits annotated to known genes are shown with labels.

Overall, we investigated the associations between leukocyte DNA methylation levels at 443,169 CpGs and 121 platelet function traits. We identified 46 CpG and platelet reactivity trait associations that survived multiple testing with FDR ≤ 0.05 ( Fig. 1 ; Table 3 ). There were not many differences between the utilized model and models adjusted for common medications (P2Y12 inhibitors, antiplatelet, anticoagulant, and antidepressant drugs) aside from small attenuations in unadjusted *p* -values. All 46 significant associations were preserved in the medication-adjusted model, which can be observed in Supplementary Table S2 .

**Table 3:** Significant associations that survived multiple testing with an FDR of ≤ 0.05

CpG	Annotated gene (in or nearby)	Gene region	CHR	Probe SNPs	Platelet agonist	Platelet assay	Platelet trait name	Beta	Standard error (SE)	*p* -Value	Multiple test adjusted *p* -Value
cg24267699	*ABO*	Intergenic	9		Ristocetin	LTA	Risto_PrimSlope	0.214	0.031	1.04E-11	4.61E-06
cg21160290	*ABO*	Intron	9		Ristocetin	LTA	Risto_PrimSlope	0.179	0.028	1.59E-10	3.51E-05
cg22535403	*ABO*	Intron	9		Ristocetin	LTA	Risto_PrimSlope	0.171	0.028	7.87E-10	1.16E-04
cg11879188	*ABO*	Intron	9		Ristocetin	LTA	Risto_PrimSlope	0.170	0.028	1.32E-09	1.46E-04
cg25020897	*CAPRIN2*	Intron	12		ADP	Flow cytometry	FI_wb_PAC1%	0.168	0.028	2.17E-09	9.64E-04
cg14285533	*LINC2848*	LINCRNA	7		ADP	LTA	ADPmid_DisAgg	0.118	0.020	5.40E-09	2.39E-03
cg22380533	*SYT5*	Intron	19	rs11672000	ADP	Flow cytometry	FI_wb_PAC1%	0.161	0.028	1.51E-08	3.35E-03
cg07762993	*KIF25-AS1*	Intergenic	6	rs78086629	Collagen	LTA	Coll_FinAgg	−0.157	0.028	2.42E-08	1.07E-02
cg19372507	*MINAR1*	Intergenic	15		ADP	Optimul	ADPecMax	0.121	0.022	4.83E-08	1.28E-02
cg10321623	*B4GALNT3*	Exon	12		ADP	Optimul	ADPecMax	0.126	0.023	5.76E-08	1.28E-02
cg10122766	*TLE6*	Intergenic	19		ADP	Flow cytometry	FI_prp_PAC1%	0.161	0.029	3.97E-08	1.66E-02
cg22380533	*SYT5*	Intron	19	rs11672000	ADP	Flow cytometry	FI_prp_PAC1%	0.158	0.029	7.49E-08	1.66E-02
cg10512202	*LIMD1*	Intron	3		Arachidonic acid	LTA	AA_AUC.std	−0.112	0.020	4.49E-08	1.99E-02
cg25385322	*FIS1*	Intron	7		Ristocetin	LTA	Risto_AUC.std	−0.162	0.030	4.67E-08	2.07E-02
cg10122766	*TLE6*	Intergenic	19		ADP	Flow cytometry	FI_prp_PAC1_Psel_DoublePos%	0.160	0.029	4.75E-08	2.11E-02
cg02936049	*ZBTB38*	Intron	3		ADP	LTA	ADPlow_AUC.std	0.190	0.035	4.83E-08	2.14E-02
cg11772020	*LINC2537*	Intergenic	6	rs79479645	Arachidonic acid	Multiplate	MPaspiAgg	−0.200	0.037	5.20E-08	2.30E-02
cg07762993	*KIF25-AS1*	Intergenic	6	rs78086629	Collagen	LTA	Coll_PrimAgg	−0.153	0.028	5.21E-08	2.31E-02
cg07762993	*KIF25-AS1*	Intergenic	6	rs78086629	Collagen	LTA	Coll_MaxAgg	−0.153	0.028	5.21E-08	2.31E-02
cg10512202	*LIMD1*	Intron	3		Arachidonic acid	LTA	Aa_MaxAgg	−0.111	0.020	5.27E-08	2.34E-02
cg10512202	*LIMD1*	Intron	3		Arachidonic acid	LTA	Aa_PrimAgg	−0.111	0.020	5.27E-08	2.34E-02
cg10512202	*LIMD1*	Intron	3		Arachidonic acid	LTA	Aa_FinAgg	−0.109	0.020	5.52E-08	2.44E-02
cg24965248	*FGF6*	Intron	12	rs71583763	Epinephrine	LTA	Epi10_SecAgg	−0.118	0.022	6.01E-08	2.66E-02
cg01078871	*FBXO33*	Intron	14		ADP	Flow cytometry	FI_wb_PAC1%	0.146	0.028	1.81E-07	2.68E-02
cg25385322	*FIS1*	Intron	7		Ristocetin	LTA	Risto_FinAgg	−0.158	0.029	8.80E-08	3.32E-02
cg23201265	*LINC2955*	LINCRNA	12		Ristocetin	LTA	Risto_FinAgg	0.147	0.028	1.93E-07	3.32E-02
cg20417128	*PXDN*	Intron	2		Ristocetin	LTA	Risto_FinAgg	0.147	0.028	2.25E-07	3.32E-02
cg04738774	*CALD1*	Intron	7		ADP	Optimul	ADPaucMean	−0.161	0.030	7.74E-08	3.43E-02
cg20732755	*PRKN*	Intron	6		ADP	LTA	ADPmid_DisAgg	−0.103	0.020	1.56E-07	3.45E-02
cg06213060	*LINC00690*	LINCRNA	3		ADP	Optimul	ADPecMax	−0.153	0.030	3.00E-07	3.56E-02
cg25287211	*CLN5*	Intron	13		ADP	Optimul	ADPecMax	−0.114	0.022	3.22E-07	3.56E-02
cg03892812	*KNDC1*	Intron	10		ADP	Optimul	ADPecMax	0.124	0.025	4.06E-07	3.60E-02
cg01294058	*CDKN1C*	Intron	11		ADP	LTA	ADPmid_DisAgg	−0.097	0.019	3.70E-07	3.63E-02
cg18905668	*GTF2H1*	Intron	11		ADP	LTA	ADPmid_DisAgg	0.124	0.025	4.34E-07	3.63E-02
cg04970158	*PLCD3*	Intron	17		ADP	LTA	ADPmid_DisAgg	0.101	0.020	4.91E-07	3.63E-02
cg20206224	*XPO5*	Intron	6		ADP	Flow cytometry	FI_wb_PAC1%	0.141	0.028	4.15E-07	3.78E-02
cg14757344	*RRP1*	Intron	21		ADP	Flow cytometry	FI_wb_PAC1%	0.143	0.028	4.27E-07	3.78E-02
cg00814244	*MYCN*	Intergenic	2		ADP	Optimul	ADPecMax	−0.108	0.022	6.51E-07	4.23E-02
cg14823535	*SUN1*	Intron	7		ADP	Optimul	ADPecMax	0.105	0.021	8.10E-07	4.23E-02
cg09249084	*WLS; GNG12-AS2*	Exon; Intron	1		ADP	Optimul	ADPecMax	0.106	0.021	8.58E-07	4.23E-02
cg25385322	*FIS1*	Intron	7		Arachidonic acid	LTA	Aa_FinAgg	−0.110	0.021	1.93E-07	4.29E-02
cg10948783	*FAM241A*	Intron	4		ADP	Optimul	ADPecMax	−0.108	0.022	1.01E-06	4.47E-02
cg19342368	*WHAMM*	Intergenic	15		ADP	Optimul	ADPaucMean	0.153	0.030	2.12E-07	4.70E-02
cg04231958	*UBE3B*	Intergenic	12	rs28384416	ADP	Flow cytometry	FI_wb_PAC1%	0.145	0.029	6.50E-07	4.80E-02
cg20417128	*PXDN*	Intron	2		Ristocetin	LTA	Risto_MaxAgg	0.147	0.028	2.19E-07	4.84E-02
cg26962595	*STARD10*	Intron	11		None	Flow cytometry	fc_wbNSEstimatePLT	−0.151	0.028	1.10E-07	4.87E-02

Note: Interpretation of platelet trait names can be found in Supplementary Table S1 .

#### *ABO* , VWF, and a Ristocetin Reactivity Trait Elastic Net

The most significant associations were located at four different CpGs within and adjacent to the *ABO* locus. Three CpGs all clustered within intron 1 approximately 600 to 750 bp from the consensus transcription start site (TSS) of *ABO* , and one CpG (cg24267699) dinucleotide was located −742/− 743 bp upstream of the consensus RefSeq TSS. The same ristocetin-based platelet reactivity trait (Risto_PrimSlope, primary wave slope for LTA ristocetin responses) was represented in these four associations, with *p* -values ranging from 1.32E-09 to 1.04E-11. All β values were in the positive effect direction (β range: 0.170–0.214, standard error (SE) range: 0.028–0.031), indicating association of increased methylation with an upregulation of ristocetin-driven platelet reactivity. When investigating the associations between VWF plasma levels and leukocyte DNA methylation, we find four signals with an FDR ≤ 0.05; all of which are associated with methylation in the four *ABO* CpGs significant in our platelet function analysis (cg11879188, cg22535403, cg24267699, cg21160290). All β were in the positive direction, ranging from 0.14 to 0.20, with *p* -values ranging from 1.52E-07 to 1.52E-10 and SE values of 0.02 to 0.03. These associations are listed in Table 4 .

**Table 4:** Significant associations between VWF plasma levels and DNA methylation that survived multiple testing with an FDR of ≤ 0.05

CpG	Annotated gene (in or nearby)	Beta	Standard error (SE)	*p* -Value	Multiple test adjusted *p* -value
cg21160290	*ABO*	0.180	0.028	1.52E-10	6.72E-05
cg24267699	*ABO*	0.202	0.032	1.72E-10	3.80E-05
cg22535403	*ABO*	0.164	0.028	4.65E-09	6.85E-04
cg11879188	*ABO*	0.149	0.028	1.52E-07	1.69E-02

As the associations for *ABO* CpGs were our strongest, and in conjunction with the confirmation that plasma VWF is also associated with *ABO* methylation, we wanted to investigate the predictive capacity of DNA methylation to estimate Risto_PrimSlope responses. This decision was made not only because of the four CpG signals in *ABO* , but also because prior work found a known association (β = − 0.415; *p* = 7.27E-09; *n* = 2,899) between Risto_PrimSlope and VO _2_ max, a measure of oxygen intake and utilization reflecting fitness level. 29 We utilized an elastic net approach using the *cv.glmnet* and *glmnet* functions in the *glmnet* R package. 32 In brief, 1,286 samples analyzed in the Risto_PrimSlope EWAS were randomly split into training ( *n* = 800) and validation ( *n* = 486) datasets. We used an adaptive penalty calculated from the EWAS summary statistics of 4,406 CpGs with a *p-* value of < 0.01; and this was used in conjunction with the standardized CpG data and Risto_PrimSlope in the training dataset in the elastic net analysis. A complete procedure is described in **Supplementary Method S1** . Based on 100 replicates to test model performance, the predictor and Risto_PrimSlope had a mean and range of: Pearson correlation of 0.78 ( *R* ^2^ = 0.61) and 0.75 to 0.82; and standardized root mean squared error (sRMSE) of 0.69 and 0.62 to 0.75. The correlation is strong and stable, while the sRMSE is moderate. For validation with the known association of Risto_PrimSlope with peak VO _2_ , we repeated the association testing using each of the Risto_PrimSlope and the predictor as an outcome. We used a *t* -test to compare peak VO _2_ effect estimates from two sets of association testing. The results did not support a statistically significant difference between the two peak VO _2_ effect estimates in all replicates. See Supplementary Fig. S1 for scatter plots of the peak VO _2_ estimates and −log10 *p* -values.

#### Other CpG to Platelet Function Associations

A single CpG within an intronic region of the *LIMD1* gene also had multiple significant associations, which were found to be associated with four highly correlated LTA AA traits ( *p* -value range: 5.52E-8–4.49E-8, all *β* = − 0.11, SE = 0.02), suggesting downregulation of platelet reactivity. Additionally, a CpG in an exon region of *SYT5* had two significant associations with correlated FC ADP traits (WB and PRP platelet-platelet adhesion via PAC-1 antibody marker, with *p* -value range: 7.49E-08–1.51E-08, all β = 0.16, SE = 0.02). The CpG within *SYT5* has a probe SNP (rs11672000) with approximately 17.1% minor allele frequency in European ancestry individuals that could have an effect on these results. In a similar vein, a single CpG located approximately 175 bp upstream of the *TLE6* TSS was found to have two significant associations with higher ADP platelet reactivity measured by FC ( *p* -value range: 4.75E-08–3.97E-08, all β = 0.16, SE = 0.02). A single CpG in intron 4 of *PXDN* also had two significant associations with higher ristocetin platelet activation ( *p* -value range: 2.25E-07–2.19E-07, all β = 0.14, SE = 0.02). Finally, a single CpG located within intron 3 of *FIS1* was associated with decreased platelet reactivity after ristocetin and AA stimulation ( *p* -value range: 1.93E-7–4.67E-8, β range: −0.10 to −0.16, SE range: 0.02–0.03). Other significant CpG and platelet reactivity associations are listed in Table 3 .

**Fig. 2:**
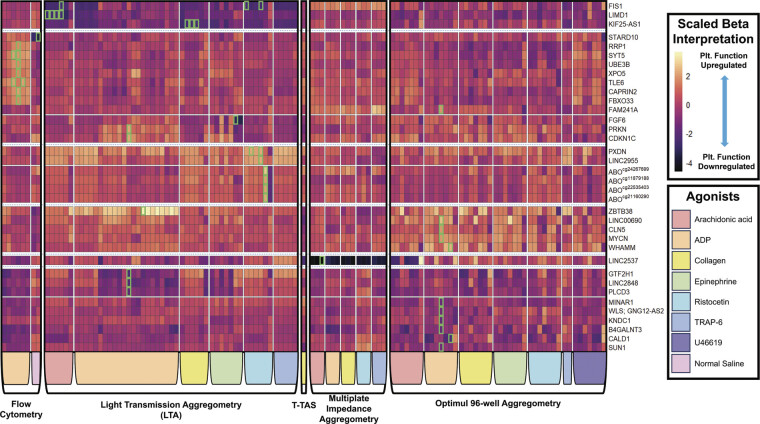
Heatmap of scaled β values across all platelet reactivity traits for 36 significant CpGs. Betas for certain traits were initially transformed in order for all positive values to reflect an upregulation in platelet function and vice versa (see Supplementary Table S1 ). Beta values were scaled using the default R function. The plot is divided vertically by assay (indicated by large brackets) and then further subdivided by agonists measured within the assay (colored subsets). CpGs are represented by rows, which were clustered into six bins using *k* -means clustering within the NbClust R package. Significant hits are shown with green boxes around individual cells.

Ristocetin, ADP, collagen and epinephrine-related platelet functional traits were represented among the 46 significant hits, with the LTA assay driving over half of the significant associations. When comparing β across all traits for the 36 significant CpGs ( Fig. 2 ), there is observable directional clustering across assays and agonists. This is clear when examining MP traits for cg11772020 (annotated to *LINC2537* ); LTA ristocetin and LTA TRAP-6 traits for cg07762933 (annotated to *KIF25-AS1* ); and hits annotated to *FIS1* and *LIMD1* .

### Megakaryocyte Epigenome Overlaps

**Fig. 3:**
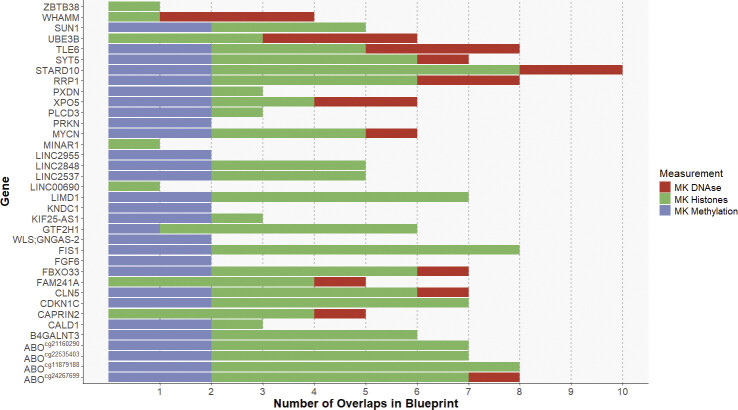
Overall Blueprint overlaps by CpG. Bar chart of the number of epigenome features overlapping between significant CpG sites and Blueprint MK sample features (methylation, histone, DNase-I sites).

To better understand the potential links between our 46 significant CpG associations with platelet reactivity, we examined epigenetic measurements in MKs from data provided by Blueprint. Of the 36 unique significant CpGs, all had at least one overlap with either MK methylation by an orthogonal technology (bisulfite sequencing), DNase-I sites reflecting open chromatin, ChIP-seq data, or a combination thereof ( Fig. 3 ).

**Fig. 4:**
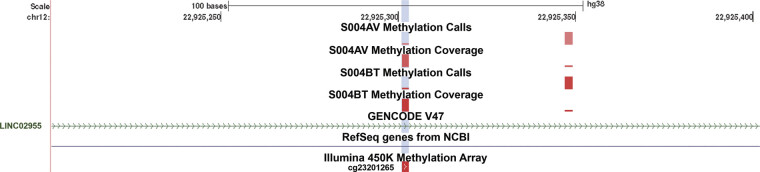
UCSC genome browser view of cg23201265 Blueprint MK methylation data calls and coverage. The CpG of interest is shown highlighted in blue. S004BT methylation calls and coverage are shown in purple. The CpG of interest and gene calls (provided by GENCODE and RefSeq via NCBI) are also shown; while assayed MK methylation data appears to align with our CpG of interest, the average methylation values in Table 3 still may reflect more than a single CpG within a measured region.

All but seven CpGs had overlapping MK methylation data in Blueprint ( Supplementary Table S3 ). A few queried regions were small enough and contained so few CpGs—specifically in the case of cg23201265 (annotated to *LINC2955* ) that the methylation coverage and calls could be aligned with the CpG of interest ( Fig. 4 ). Other similar instances include an overlapping region of cg04738774 (annotated to *CALD1* ) containing six CpGs; and two overlapping regions with cg14285533 (annotated to *LINC2848* ) which contained 10 and 11 CpGs, respectively. The overlapping regions of three of the four CpGs annotated to intron 1 of *ABO* match, as those CpGs are clustered within a few thousand bp. However, other significant CpGs we measured by Illumina arrays corresponded to wider regions labeled as hyper or hypomethylated via bisulfite sequencing in Blueprint, and we could not co-localize overlapping CpG calls.

The level of methylation in all these regions was a mix of both hyper and hypomethylated calls. Of the *ABO* queries, the three intronic sites were hypomethylated, while the single CpG (cg11879188) located upstream from the TSS was hypermethylated in MKs. The mixture in correspondence with Blueprint regional methylation with an orthogonal technology is reflected by our findings when we conducted 1,000 random iterations of sets of 36 CpGs from among the 443,169 tested. We observed no apparent enrichment of overlap with MK methylated sites, hypomethylated sites, or hypermethylated sites.

Thirteen of the 36 significant CpGs had overlapping DNase-I hypersensitivity activity calls ( Supplementary Table S4 ). Of these, six CpGs had overlapping MK DNase activity across both samples (or both experiments for sample C006NS and measures from S004BT, present in three of those six CpGs). As all DNase query regions were 150 bp in size, reflective of peak calling around enzyme activity, it is likely that it is localized to the immediate region of the CpGs of interest. In total, there were 22 overlaps of our CpGs with DNase sample peaks. Remarkably, this proved to be highly enriched relative to 1,000 random iterations of sets of 36 significant CpGs among the 443,169 we tested ( Supplemental Fig. S2 ).

Unlike DNase sites, ChIP-seq histone marker sites varied greatly in size, often being several thousand bp ( Supplementary Table S5 ). All but five of our 36 significant CpGs showed matching ChIP-seq calls, with many CpGs having calls across multiple samples and histone modifications. In total, there were 105 CpG to ChIP-seq region alignments in MKs. In examining random sets of 36 CpGs among our test set, it was highly unusual to observe this many CpG-to-ChIP-seq alignments, suggesting significant enrichment. Overall, our results suggested that our platelet reactivity-associated CpG sites are highly enriched for MK epigenetic marks (DNase-I sites, histone sites) but not for methylation patterns measured by the orthogonal bisulfate sequencing approach.

**Table 5:** Significant associations between leukocyte DNA methylation and platelet RNA transcript levels

CpG	RNA transcript ID	Beta	Standard error (SE)	*p* -Value	CpG location notes
cg22535403	ABO_ENSG00000175164.15	−0.571	0.057	1.69E-23	In intron 1, 0.5 kb from TSS
cg21160290	ABO_ENSG00000175164.15	−0.534	0.059	1.04E-19	In intron 1, 0.5 kb from TSS
cg11879188	ABO_ENSG00000175164.15	−0.367	0.064	1.20E-08	In intron 1, 0.5 kb from TSS
cg24267699	ABO_ENSG00000175164.15	−0.370	0.065	1.26E-08	In upstream promoter, 0.5 kb from TSS
cg10512202	LIMD1_ENSG00000144791.10	0.315	0.067	2.25E-06	In intron 1, 14 kb from TSS

**Fig. 5:**
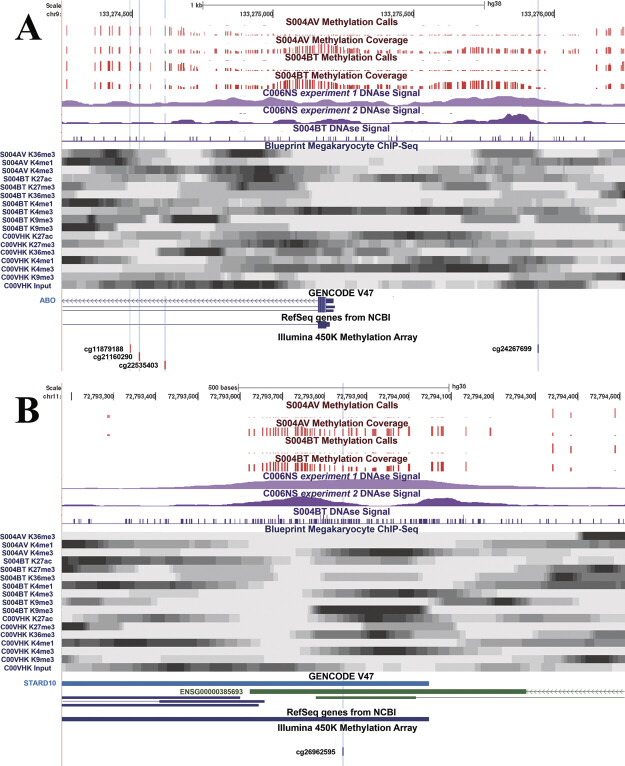
( **A** ) UCSC genome browser view of all CpGs annotated to *ABO* with Blueprint MK methylation calls and coverage; DNase activity across two samples (with one repeat); and ChIP-seq data across three samples and multiple histone modifications. The CpGs of interest are highlighted using a vertical blue bar. ( **B** ) UCSC genome browser view of cg26962595 (annotated to *STARD10* ) with Blueprint MK methylation calls and coverage; DNase activity across two samples (with one repeat); and ChIP-seq data across three samples and multiple histone modifications. The CpG of interest is highlighted using a vertical blue bar.

The patterns of MK epigenetic regulation combined with our trait associations and literature search reveal several interesting insights. For example, of the significant CpGs, 9 possessed one or more overlaps with all three categories of MK data: methylation, DNase, and ChIP-seq. Notable examples of this included one CpG site (cg24267699) annotated to *ABO* ( Fig. 5A ), and a CpG site (cg26962595) annotated to *STARD10* ( Fig. 5B ), as well as seven others displayed in Fig. 3 . This suggests high levels of functional epigenetic regulation at these sites.

### Platelet RNA to Leukocyte DNA Methylation Associations

The leukocyte DNA methylation and platelet RNA transcript level associations identified five CpGs with a *p* -value of ≤ 0.05. All of these associations are in cis, where cis is defined as one megabase from the forward strand TSS or one megabase from the reverse strand end site. Notably, all four significant CpGs annotated to *ABO* from the DNA methylation and platelet function analysis were not only significant here as well but also demonstrated downregulated levels of RNA transcript expression. These CpGs were also all approximately 500 bp from the TSS, located within intron 1 of *ABO* , save for cg24267699, which was located −742/− 743 upstream of the TSS ( Table 5 ). The other significant result was cg105212202 (annotated to *LIMD1* ). This CpG is located within the first intron of *LIMD1* , and the β value is in the positive effect direction, indicating a role of leukocyte DNA methylation in the ultimate upregulation of *LIMD1* gene expression in platelets. While we could not directly test MK methylation and epigenetic associations with platelet RNA expression here, we can make an inference that alterations at these sites in haemopoietic stem cells, MKs, or other cell lineages may influence gene expression levels and the roles of these genes in platelet functions.

As an additional point, we were curious about protein-level expression of *LIMD1* , since it has not been widely studied in platelets. To validate relative protein expression of *LIMD1* in platelets, we utilized isobaric labels and Tandem Mass Tags mass spectrometry data in washed platelets from 30 age and sex-matched samples from participants enrolled in National Institutes of Health biosampling studies, using methods described in **Supplementary Method S2** . We found reporter ion intensities with an average reporter ion intensity of 388,548 and median reporter ion intensity of 382,476 across all samples, confirming relative protein-level expression of *LIMD1* in platelets. Furthermore, prior work by Burkhart et al to characterize the platelet proteome in four donor samples confirms *LIMD1* is expressed in human platelets with around 810 copies per platelet at a reported 100% confidence level. 33

## Discussion

Our analysis revealed several significant associations between platelet function phenotypes and DNA methylation. Foremost, we identified a few genes annotated to significant CpGs known to be implicated in platelet function. One such example is *ABO* , which regulates vWF and is a ristocetin agglutination determinant via the GPIb-V-IX receptor complex. 34 Of the significant *ABO* -annotated CpGs, three had overlapping hypomethylation measures and histone modifications in MKs, with cg24267699 having overlapping DNase, histone modifications, and hypomethylation measures in MKs. Furthermore, all four of the *ABO* -annotated CpGs were associated with platelet *ABO* transcript levels. Notably, *ABO* blood group status has been linked to differential methylation in the promoter, including at cg24267699. 35 *ABO* is known to regulate the levels of many important protein targets in blood, and previous studies showed that differential methylation at cg24267699 may be an important mediator of key proteins vWF, F8, and soluble E-selectin. 35 36 Our analysis of vWF plasma levels and leukocyte DNA methylation also further affirmed that methylation of *ABO* in WBCs is associated with vWF levels, which solidifies the potential regulatory role of *ABO* methylation in mediating plasma protein expression. In a large epigenome-wide scan for causal inference associations, cg24267699 methylation was shown to associate with increased coronary heart disease risk (OR: 2.89, Mendelian randomization *p* < 1.34E-06). 18 Ours is the first study to link *ABO* methylation to putative MK epigenetic regulation, measured platelet gene expression and platelet reactivity phenotypes. This suggests that blood group status influences *ABO* methylation, *ABO* expression and the expression of multiple target proteins, and the effects on thrombosis are at least partially mediated—directly or indirectly—via altered platelet reactivity.

Another example of significant CpGs involved in platelet function is cg20417128 (annotated to *PXDN* ), which expresses the protein peroxidasin, shown to be critical in crosslinking collagen IV promoters in basement membranes. *PXDN* has an additional role in heart and vessel remodeling, with a *PXDN* antagonist (PXDNL) detected in failing myocardium. 37 38 Both significant CpGs within the *PXDN* locus were significantly associated with ristocetin traits; and as extracellular matrix is exposed at sites of vascular injury, vWF interfaces with platelets to initiate thrombus formation, being a required cofactor of ristocetin-induced aggregation. 39 To elaborate on this, a 2016 study 40 identified cg20417128 as a site of differential methylation when comparing asthmatic to nonasthmatic endobronchial airway epithelial cells. As airway remodeling is a feature of allergic asthma, which is partially characterized by epithelial injury, 41 it stands to reason that epigenetic regulation of *PXDN* may contribute to this disease etiology as well, though whether this is via effects on platelet function remains uncertain. Additionally, calcium-dependent secretion of Weibel–Palade bodies, which contain vWF, inflammatory mediators, and growth factors, is known to be regulated by synaptotagmin-5 ( *SYT5* ) in endothelial cells. 42 Other synaptotagmin isoforms and synaptotagmin-like proteins 43 are thought to regulate similar granule release processes in platelets, 44 though to our knowledge this has not yet been studied for *SYT5* .

Yet another cornerstone of platelet biology is the extensive morphological change undergone through the lifespans of MKs and platelets. This facilitates proplatelet generation and platelet release (from MKs), and platelet granule secretion, integrin activation, aggregation, adhesion, and contractile processes, among others. To support these changes, a variety of cytoskeletal elements are critically involved. 45 While the molecular mechanisms behind these changes are complex and involve a multitude of proteins both translated in platelets and inherited from MKs, our results may provide general insight into the role of methylation in regulating these processes. For instance, *TLE6* is known to interact with F-actin in embryonic development, and defects lead to an early embryonic lethal phenotype; mutant *TLE6* exhibits impaired binding potential for the cell cycle regulator PKA. 46 47 While *TLE6* is known to be highly abundant in reproductive cells, it is also highly expressed in epithelial cells during pancreas development and promotes neurogenesis by acting as an antagonist to the gene-repressing interactions of TLE1 and BF-1. 48 49 As Blueprint data suggests the *TLE6* region is an active regulatory region in MKs ( Supplementary Table S3 ), with measurable levels of DNase activity, MK methylation, and multiple histone modifications, it may serve a similar role in the differentiation process of MKs, significantly impacting downstream platelet function. Similarly, *STARD10* has a phospholipid-binding function, influencing membrane lipid composition. 50 Another study noted that *STARD10* knockout β islet cells possessed altered granule composition and levels of basal secretion of proinsulin, and synaptotagmin proteins were also differentially expressed in the *STARD10* knockout β islet cells. 51 While this mechanism has not been investigated in platelets, *STARD10* data in Blueprint MK measurements suggest this locus is a highly active regulatory region in MKs and may contribute to platelet function via its known roles in membrane and granule composition.

Furthermore, cytoskeletal motility is critical for platelet function. *LIMD1* has a mechanically triggered role in assembling focal adhesion for the purpose of transduction and cell migration, in addition to regulating cell spreading, contractility, and durotaxis, 52 53 key processes in platelets. In addition, *LIMD1* DNA methylation in leukocytes was associated with increased platelet RNA transcript levels. Our results also confirm *LIMD1* protein expression in platelets. Thus, altered *LIMD1* expression in platelets may influence platelet spreading and activation in response to AA. Several other examples of proteins critical for cell architecture were observed, including *CALD1* , which is implicated in actin cytoskeletal rearrangement. 54 In the Golgi apparatus, WHAMM is known to regulate membrane dynamics at the microtubule-actin interface, critical to organelle structure. 55 A facet of these processes is the robust energy requirements necessary for the metabolism of a platelet, and mitochondria—which are enriched in platelets—are chief in fulfilling this responsibility. 56 *FIS1* partially controls mitochondrial fission, which is an important regulatory element of mitochondrial function. 57 While it is unknown how methylation may drive the expression of these genes throughout the lifespan of a differentiating myeloid cell, at a locus level in a few samples from Blueprint data, all appear to be methylated in MKs. Taken together, this suggests that methylation may drive expression patterns for some of the vital regulatory mechanisms integral to platelet shape change and dynamics in both MKs and platelets. This activity may ultimately influence the downstream reactivity of circulating platelets as suggested by our population-level associations in a large sample.

In addition, we observed associations at loci encoding multiple transcription factors, including *ZBTB38* , which is directly involved in the regulation of DNA methylation and plays a role in transcription regulation. 58 59 Another associated gene locus is *CAPRIN2* , known to promote activation of the canonical Wnt signaling transduction pathway to regulate transcription, in addition to having other posttranscriptional regulatory roles in the face of osmotic stress. 60 61 *WLS* is also involved in the regulation of the Wnt signaling pathway. 62 In MKs, Wnt signaling is known to modulate proliferation, maturation, and proplatelet formation. Both canonical and noncanonical Wnt signaling pathways have diverse roles in the regulation of MK gene expression, though these mechanisms are not fully understood. 63 Recently, several gene loci for immune thrombocytopenia (ITP) were identified in a GWAS, implicating genes ( *NAV2* , *NKD1* , *CCDC85A* , *GRIP1* ) that affect Wnt/β-catenin signaling in ITP disease risk. 64 In platelets, the Wnt signaling pathway may have a functional role, affecting activation, secretion, aggregation, and adhesion. Prior work suggests the Wnt3a ligand may act to block ADP-induced shape change, 65 which aligns with *ZBTB38* , *CAPRIN2* , and *WLS* CpGs being significantly associated with ADP-based functional traits across multiple assays.

The overall enrichment of epigenetic activity at our 36 significant CpGs within the Blueprint samples suggests that many of our CpGs are near or within active MK regulatory regions. We may be able to surmise that this enrichment indicates that DNA methylation of these sites in MKs contributes to the development of functioning platelets with differing attributes in respect to activating stimuli. However, identifying the specific underlying mechanisms would require detailed studies in MKs or developing lineage cells, which is beyond the scope of this study. While we are limited in interpreting our significant results based on the literature and known gene functions, this study nonetheless extends the knowledge of DNA methylation influences on platelet functions, including cell motility, adhesion, and energy metabolism, adding to prior work on single loci. 9 10

Our study has several limitations to note. Our methylation data and platelet function data were measured at different exams approximately 7 years apart, which may not account for changes in methylation or platelet function between those two time periods. This is in addition to other lifestyle alterations like a change in smoking status or weight, which may also affect our results. However, some studies suggest certain CpGs have stable methylation over periods of several years to even decades, 66 67 though further research into the stability of the significant CpGs in our study is needed. Additionally, it is noted that leukocyte and platelet function are intertwined, and that platelets play a vital role in the innate immune response. Activated platelets may directly interact with leukocytes in inflammatory conditions, and platelets have been demonstrated to recruit neutrophils to generate reactive oxygen species or generate NETs, which are antibacterial webs of extracellular DNA and histones for pathogen clearance. 68 69 Inversely, leukocytes also regulate platelet functional responses via release of activating proteins, such as coagulation factors. 70 It is also known that leukocytes may contribute to the etiology of thrombosis via interactions with platelets. 71 Ultimately, these interactions may affect ex vivo platelet functional measurements, as the formation of leukocyte-platelet aggregates and release of mediators influences platelet behavior. Implementation of measurements of leukocyte function into statistical modeling may alleviate these concerns in future studies. Furthermore, most of the participants in the FHS are mainly of European ancestry, which may limit the generalizability of our findings to other populations. Regardless, our study also includes a large sample, which provides statistical power for our analysis compared with prior research, including a large depth of platelet functional profiling across a spectrum of assays and agonists relative to prior studies on DNA methylation and platelet function.

The aforementioned characteristics of this dataset enabled an exploration of a DNA methylation-based predictor model, especially considering the relative rarity of platelet function data. Our predictor had a high correlation with moderate sRMSE with Risto_PrimSlope, and the validation analysis results showed no difference in peak VO2 effect from the two sets of analyses. We therefore think the predictor performed reasonably well and extending the prediction to other platelet function traits deserves deeper exploration. However, it is worth noting that while the FHS is the largest cohort with this depth of platelet function data, the sample size is still limited, especially when split into training and validation samples.

Overall, we found dozens of statistically significant epigenome-wide links between platelet function and leukocyte DNA methylation, in the first study to conduct such an analysis. The results indicate that several important platelet pathways and processes, including actin cytoskeletal rearrangement and spreading, mitochondrial function, Wnt//β-catenin signaling, vesicle secretion and cargo regulation, and vWF-related activity, may be influenced by site-specific epigenetic regulation in MK blood lineage cells. This suggests that further epigenetic studies of MKs in large population samples or in differentiated cell lines, such as induced pluripotent stem cells or CD34+ hematopoietic stem cells, may yield further insights into key drivers of platelet development and function.
