# Supplementary material for: DNA Methylation Variation in Blood Cells may Impact Platelet Function
Source: TH Open. 2026 Jun 19;10:a28839068. doi: 10.1055/a-2883-9068 (PMC13289646; doi:10.1055/a-2883-9068)
Supplement: Supplementary file 1 — Supplementary Material [file 10-1055-a-2883-9068_28994137.pdf]

Thieme

Supplementary Table 1: Platelet function variable definitions.

Analysis Output Variable	Full Description	Platform	Cell Type	Agonist	Concentrations	Positive Beta Interpretation
AA_AUC.std	Arachidonic acid (AUC)	LTA	PRP ****	Arachidonic acid	500 µg/mL	↑ platelet reactivity
Aa_FinAgg	Arachidonic acid final % aggregation	LTA	PRP ****	Arachidonic acid	500 µg/mL	↑ platelet reactivity
Aa_LagTim	Lag time (s) to arachidonic acid	LTA	PRP ****	Arachidonic acid	500 µg/mL	↓ platelet reactivity
Aa_MaxAgg	Arachidonic acid maximal % aggregation	LTA	PRP ****	Arachidonic acid	500 µg/mL	↑ platelet reactivity
Aa_PrimAgg	Arachidonic acid primary wave % aggregation	LTA	PRP ****	Arachidonic acid	500 µg/mL	↑ platelet reactivity
Aa_PrimSlope	Arachidonic acid primary wave slope	LTA	PRP ****	Arachidonic acid	500 µg/mL	↑ platelet reactivity
AAAgg20	Arachidonic acid concentration needed to reach 20% aggregation	Optimul *	PRP ****	Arachidonic acid	0.03, 0.06, 0.11, 0.19, 0.33, 0.57, 1.0 mM	↓ platelet reactivity
AAAgg40	Arachidonic acid concentration needed to reach 40% aggregation	Optimul *	PRP ****	Arachidonic acid	0.03, 0.06, 0.11, 0.19, 0.33, 0.57, 1.0 mM	↓ platelet reactivity
AAaucMean	Arachidonic acid (AUC) across concentration range	Optimul *	PRP ****	Arachidonic acid	0.03, 0.06, 0.11, 0.19, 0.33, 0.57, 1.0 mM	↑ platelet reactivity
AAec50	Arachidonic acid concentration needed to reach 50% aggregation	Optimul *	PRP ****	Arachidonic acid	0.03, 0.06, 0.11, 0.19, 0.33, 0.57, 1.0 mM	↓ platelet reactivity
AAecMax	Arachidonic acid concentration needed to reach maximal aggregation	Optimul *	PRP ****	Arachidonic acid	0.03, 0.06, 0.11, 0.19, 0.33, 0.57, 1.0 mM	↓ platelet reactivity
AAeMax	Arachidonic acid maximal % aggregation (Optimul)	Optimul *	PRP ****	Arachidonic acid	0.03, 0.06, 0.11, 0.19, 0.33, 0.57, 1.0 mM	↑ platelet reactivity
AAslope	Arachidonic acid slope across concentration range	Optimul *	PRP ****	Arachidonic acid	0.03, 0.06, 0.11, 0.19, 0.33, 0.57, 1.0 mM	↑ platelet reactivity
ADPAgg20	ADP concentration needed to reach 20% aggregation	Optimul *	PRP ****	ADP	0.005, 0.02, 0.10, 0.44, 1.98, 8.89, 40 µM	↓ platelet reactivity
ADPAgg40	ADP concentration needed to reach 40% aggregation	Optimul *	PRP ****	ADP	0.005, 0.02, 0.10, 0.44, 1.98, 8.89, 40 µM	↓ platelet reactivity
ADPaucMean	ADP (AUC) across concentration range	Optimul *	PRP ****	ADP	0.005, 0.02, 0.10, 0.44, 1.98, 8.89, 40 µM	↑ platelet reactivity

ADPec50	ADP concentration needed to reach 50% aggregation	Optimul *	PRP ****	ADP	0.005, 0.02, 0.10, 0.44, 1.98, 8.89, 40 µM	↓ platelet reactivity
ADPecMax	ADP concentration needed to reach maximal aggregation	Optimul *	PRP ****	ADP	0.005, 0.02, 0.10, 0.44, 1.98, 8.89, 40 µM	↓ platelet reactivity
ADPeMax	ADP maximal % aggregation (Optimul)	Optimul *	PRP ****	ADP	0.005, 0.02, 0.10, 0.44, 1.98, 8.89, 40 µM	↑ platelet reactivity
ADPhigh_AUC	ADP (5.71 µM) (AUC)	LTA	PRP ****	ADP	5.71 µM	↑ platelet reactivity
ADPhigh_DisAgg	ADP (5.71 µM) % disaggregation	LTA	PRP ****	ADP	5.71 µM	↓ platelet reactivity
ADPhigh_FinAgg	ADP (5.71 µM) final % aggregation	LTA	PRP ****	ADP	5.71 µM	↑ platelet reactivity
ADPhigh_MaxAgg	ADP (5.71 µM) maximal % aggregation (LTA)	LTA	PRP ****	ADP	5.71 µM	↑ platelet reactivity
ADPhigh_PrimAgg	ADP (5.71 µM) primary wave % aggregation	LTA	PRP ****	ADP	5.71 µM	↑ platelet reactivity
ADPhigh_PrimSlope	ADP (5.71 µM) primary wave slope	LTA	PRP ****	ADP	5.71 µM	↑ platelet reactivity
ADPlow_AUC	ADP (0.95 µM) (AUC)	LTA	PRP ****	ADP	0.95 µM	↑ platelet reactivity
ADPlow_DisAgg	ADP (0.95 µM) % disaggregation	LTA	PRP ****	ADP	0.95 µM	↓ platelet reactivity
ADPlow_FinAgg	ADP (0.95 µM) final % aggregation	LTA	PRP ****	ADP	0.95 µM	↑ platelet reactivity
ADPlow_MaxAgg	ADP (0.95 µM) maximal % aggregation (LTA)	LTA	PRP ****	ADP	0.95 µM	↑ platelet reactivity
ADPlow_PrimAgg	ADP (0.95 µM) primary wave % aggregation	LTA	PRP ****	ADP	0.95 µM	↑ platelet reactivity
ADPlow_PrimSlope	ADP (0.95 µM) primary wave slope	LTA	PRP ****	ADP	0.95 µM	↑ platelet reactivity
ADPlow_SecAgg	ADP (0.95 µM) concentration secondary wave % aggregation	LTA	PRP ****	ADP	0.95 µM	↑ platelet reactivity
ADPlow_SecSlp	ADP (0.95 µM) concentration secondary wave slope	LTA	PRP ****	ADP	0.95 µM	↑ platelet reactivity
ADPmid_AUC	ADP (1.82 µM) (AUC)	LTA	PRP ****	ADP	1.82 µM	↑ platelet reactivity

ADPmid_DisAgg	ADP (1.82 µM) % disaggregation	LTA	PRP ****	ADP	1.82 µM	↓ platelet reactivity
ADPmid_FinAgg	ADP (1.82 µM) final % aggregation	LTA	PRP ****	ADP	1.82 µM	↑ platelet reactivity
ADPmid_MaxAgg	ADP (1.82 µM) maximal % aggregation (LTA)	LTA	PRP ****	ADP	1.82 µM	↑ platelet reactivity
ADPmid_PrimAgg	ADP (1.82 µM) primary wave % aggregation	LTA	PRP ****	ADP	1.82 µM	↑ platelet reactivity
ADPmid_PrimSlope	ADP (1.82 µM) primary wave slope	LTA	PRP ****	ADP	1.82 µM	↑ platelet reactivity
ADPmid_SecAgg	ADP (1.82 µM) concentration secondary wave % aggregation	LTA	PRP ****	ADP	1.82 µM	↑ platelet reactivity
ADPmid_SecSlp	ADP (1.82 µM) concentration secondary wave slope	LTA	PRP ****	ADP	1.82 µM	↑ platelet reactivity
ADPslope	ADP slope across concentration range	Optimul *	PRP ****	ADP	0.005, 0.02, 0.10, 0.44, 1.98, 8.89, 40 µM	↑ platelet reactivity
Coll_AUC.std	Collagen (AUC)	LTA	PRP ****	Collagen	190 µg/mL	↑ platelet reactivity
Coll_FinAgg	Collagen final % aggregation	LTA	PRP ****	Collagen	190 µg/mL	↑ platelet reactivity
Coll_LagTim	Lag time (s) to collagen	LTA	PRP ****	Collagen	190 µg/mL	↓ platelet reactivity
Coll_MaxAgg	Collagen maximal % aggregation (LTA)	LTA	PRP ****	Collagen	190 µg/mL	↑ platelet reactivity
Coll_PrimAgg	Collagen primary wave % aggregation	LTA	PRP ****	Collagen	190 µg/mL	↑ platelet reactivity
Coll_PrimSlope	Collagen primary wave slope	LTA	PRP ****	Collagen	190 µg/mL	↑ platelet reactivity
CollAgg20	Collagen concentration needed to reach 20% aggregation	Optimul *	PRP ****	Collagen	0.01, 0.04, 0.16, 0.62, 2.5, 10, 40 µg/mL	↓ platelet reactivity
CollAgg40	Collagen concentration needed to reach 40% aggregation	Optimul *	PRP ****	Collagen	0.01, 0.04, 0.16, 0.62, 2.5, 10, 40 µg/mL	↓ platelet reactivity
CollaucMean	Collagen (AUC) across concentration range	Optimul *	PRP ****	Collagen	0.01, 0.04, 0.16, 0.62, 2.5, 10, 40 µg/mL	↑ platelet reactivity
Collec50	Collagen concentration needed to reach 50% aggregation	Optimul *	PRP ****	Collagen	0.01, 0.04, 0.16, 0.62, 2.5, 10, 40 µg/mL	↓ platelet reactivity

CollecMax	Collagen concentration needed to reach maximal aggregation	Optimul *	PRP ****	Collagen	0.01, 0.04, 0.16, 0.62, 2.5, 10, 40 µg/mL	↓ platelet reactivity
ColleMax	Collagen maximal % aggregation (Optimul)	Optimul *	PRP ****	Collagen	0.01, 0.04, 0.16, 0.62, 2.5, 10, 40 µg/mL	↑ platelet reactivity
Collslope	Collagen slope across concentration range	Optimul *	PRP ****	Collagen	0.01, 0.04, 0.16, 0.62, 2.5, 10, 40 µg/mL	↑ platelet reactivity
Epi10_AUC.std	Epinephrine (AUC)	LTA	PRP ****	Epinephrine	100 µM	↑ platelet reactivity
Epi10_FinAgg	Epinephrine final % aggregation	LTA	PRP ****	Epinephrine	100 µM	↑ platelet reactivity
Epi10_MaxAgg	Epinephrine maximal % aggregation (LTA)	LTA	PRP ****	Epinephrine	100 µM	↑ platelet reactivity
Epi10_PrimAgg	Epinephrine primary wave % aggregation	LTA	PRP ****	Epinephrine	100 µM	↑ platelet reactivity
Epi10_PrimSlope	Epinephrine primary wave slope	LTA	PRP ****	Epinephrine	100 µM	↑ platelet reactivity
Epi10_SecAgg	Epinephrine secondary wave % aggregation	LTA	PRP ****	Epinephrine	100 µM	↑ platelet reactivity
Epi10_SecSlp	Epinephrine secondary wave slope	LTA	PRP ****	Epinephrine	100 µM	↑ platelet reactivity
EpiAgg20	Epinephrine concentration needed to reach 20% aggregation	Optimul *	PRP ****	Epinephrine	0.0004, 0.001, 0.01, 0.06, 0.33, 1.82, 10 µM	↓ platelet reactivity
EpiAgg40	Epinephrine concentration needed to reach 40% aggregation	Optimul *	PRP ****	Epinephrine	0.0004, 0.001, 0.01, 0.06, 0.33, 1.82, 10 µM	↓ platelet reactivity
EpiaucMean	Epinephrine (AUC) across concentration range	Optimul *	PRP ****	Epinephrine	0.0004, 0.001, 0.01, 0.06, 0.33, 1.82, 10 µM	↑ platelet reactivity
Epiec50	Epinephrine concentration needed to reach 50% aggregation	Optimul *	PRP ****	Epinephrine	0.0004, 0.001, 0.01, 0.06, 0.33, 1.82, 10 µM	↓ platelet reactivity
EpiecMax	Epinephrine concentration needed to reach maximal aggregation	Optimul *	PRP ****	Epinephrine	0.0004, 0.001, 0.01, 0.06, 0.33, 1.82, 10 µM	↓ platelet reactivity
EpieMax	Epinephrine maximal % aggregation (Optimul)	Optimul *	PRP ****	Epinephrine	0.0004, 0.001, 0.01, 0.06, 0.33, 1.82, 10 µM	↑ platelet reactivity
Epislope	Epinephrine slope across concentration range	Optimul *	PRP ****	Epinephrine	0.0004, 0.001, 0.01, 0.06, 0.33, 1.82, 10 µM	↑ platelet reactivity
fc_prp_nsPLT_estimated	PRP platelet count	Flow cytometry	PRP ****	None	Normal saline	↑ platelet reactivity

fc_wbNSEstimatePLT	WB platelet count (estimated from volume to reach 10,000 platelets)	Flow cytometry	Whole blood ^	None	Normal saline	↑ platelet reactivity
FI_prp_PAC1 _Psel_DoublePos%	Percent PAC1+ and CD62P+ positive platelets (PRP)	Flow cytometry	PRP ****	ADP	20 µM	↑ platelet reactivity
FI_prp_PAC1%	Percent PAC1+ positive platelets (PRP)	Flow cytometry	PRP ****	ADP	20 µM	↑ cell count
FI_prp_Psel%	Percent CD62P+ positive platelets (PRP)	Flow cytometry	PRP ****	ADP	20 µM	↑ platelet reactivity
FI_wb_PAC1%	Percent PAC1+ positive platelets (WB)	Flow cytometry	Whole blood ^	ADP	20 µM	↑ platelet reactivity
FI_wb_FAC1Psel _DoublePos%	Percent PAC1+ and CD62P+ positive platelets (WB)	Flow cytometry	Whole blood ^	ADP	20 µM	↑ platelet reactivity
FI_wb_PSel%	Percent CD62P+ positive platelets (WB)	Flow cytometry	Whole blood ^	ADP	20 µM	↑ cell count
MPadpAgg	ADP impedance aggregation	Multiplate **	Whole blood ^	ADP	3.19 µM	↑ platelet reactivity
MPadpAUC	ADP impedance AUC	Multiplate **	Whole blood ^	ADP	3.19 µM	↑ platelet reactivity
MPadpVel	ADP impedance velocity	Multiplate **	Whole blood ^	ADP	3.19 µM	↑ platelet reactivity
MPaspiAgg	Arachidonic acid impedance aggregation	Multiplate **	Whole blood ^	Arachidonic acid	0.5 mM	↑ platelet reactivity
MPaspiAUC	Arachidonic acid impedance AUC	Multiplate **	Whole blood ^	Arachidonic acid	0.5 mM	↑ platelet reactivity
MPaspiVel	Arachidonic acid impedance velocity	Multiplate **	Whole blood ^	Arachidonic acid	0.5 mM	↑ platelet reactivity
MPcolAgg	Collagen impedance aggregation	Multiplate **	Whole blood ^	Collagen	61.0 µg/mL	↑ platelet reactivity
MPcolAUC	Collagen impedance AUC	Multiplate **	Whole blood ^	Collagen	61.0 µg/mL	↑ platelet reactivity
MPcolVel	Collagen impedance velocity	Multiplate **	Whole blood ^	Collagen	61.0 µg/mL	↑ platelet reactivity
MPristoAgg	Ristocetin impedance aggregation	Multiplate **	Whole blood ^	Ristocetin	1.15 mg/mL	↑ platelet reactivity
MPristoAUC	Ristocetin impedance AUC	Multiplate **	Whole blood ^	Ristocetin	1.15 mg/mL	↑ platelet reactivity

MPristoVel	Ristocetin impedance velocity	Multiplate **	Whole blood ^	Ristocetin	1.15 mg/mL	↑ platelet reactivity
MPtrapAgg	TRAP-6 impedance aggregation	Multiplate **	Whole blood ^	TRAP-6	4.48 µM	↑ platelet reactivity
MPtrapAUC	TRAP-6 impedance AUC	Multiplate **	Whole blood ^	TRAP-6	4.48 µM	↑ platelet reactivity
MPtrapVel	TRAP-6 impedance velocity	Multiplate **	Whole blood ^	TRAP-6	4.48 µM	↑ platelet reactivity
Risto_AUC	Ristocetin (AUC)	LTA	PRP ****	Ristocetin	1.5 mg/mL	↑ platelet reactivity
Risto_DisAgg	Ristocetin % disaggregation	LTA	PRP ****	Ristocetin	1.5 mg/mL	↓ platelet reactivity
Risto_FinAgg	Ristocetin final % aggregation	LTA	PRP ****	Ristocetin	1.5 mg/mL	↑ platelet reactivity
Risto_MaxAgg	Ristocetin maximal % aggregation (LTA)	LTA	PRP ****	Ristocetin	1.5 mg/mL	↑ platelet reactivity
Risto_PrimAgg	Ristocetin primary wave % aggregation	LTA	PRP ****	Ristocetin	1.5 mg/mL	↑ platelet reactivity
Risto_PrimSlope	Ristocetin primary wave slope	LTA	PRP ****	Ristocetin	1.5 mg/mL	↑ platelet reactivity
RistoAgg20	Ristocetin concentration needed to reach 20% aggregation	Optimul *	PRP ****	Ristocetin	0.14, 0.24, 0.43, 0.75, 1.31, 2.29, 4 mg/mL	↓ platelet reactivity
RistoAgg40	Ristocetin concentration needed to reach 40% aggregation	Optimul *	PRP ****	Ristocetin	0.14, 0.24, 0.43, 0.75, 1.31, 2.29, 4 mg/mL	↓ platelet reactivity
RistoaucMean	Ristocetin (AUC) across concentration range	Optimul *	PRP ****	Ristocetin	0.14, 0.24, 0.43, 0.75, 1.31, 2.29, 4 mg/mL	↑ platelet reactivity
Ristoec50	Ristocetin concentration needed to reach 50% aggregation	Optimul *	PRP ****	Ristocetin	0.14, 0.24, 0.43, 0.75, 1.31, 2.29, 4 mg/mL	↓ platelet reactivity
RistoecMax	Ristocetin concentration needed to reach maximal aggregation	Optimul *	PRP ****	Ristocetin	0.14, 0.24, 0.43, 0.75, 1.31, 2.29, 4 mg/mL	↓ platelet reactivity
RistoeMax	Ristocetin maximal % aggregation (Optimul)	Optimul *	PRP ****	Ristocetin	0.14, 0.24, 0.43, 0.75, 1.31, 2.29, 4 mg/mL	↑ platelet reactivity
Ristoslope	Ristocetin slope across concentration range	Optimul *	PRP ****	Ristocetin	0.14, 0.24, 0.43, 0.75, 1.31, 2.29, 4 mg/mL	↑ platelet reactivity
Trap_AUC	TRAP-6 (AUC)	LTA	PRP ****	TRAP-6	0.67 mM	↑ platelet reactivity

Trap_FinAgg	TRAP-6 final % aggregation	LTA	PRP ****	TRAP-6	0.67 mM	↑ platelet reactivity
Trap_MaxAgg	TRAP-6 maximal % aggregation (LTA)	LTA	PRP ****	TRAP-6	0.67 mM	↑ platelet reactivity
Trap_PrimAgg	TRAP-6 primary wave % aggregation	LTA	PRP ****	TRAP-6	0.67 mM	↑ platelet reactivity
Trap_PrimSlope	TRAP-6 primary wave slope	LTA	PRP ****	TRAP-6	0.67 mM	↑ platelet reactivity
TRAP6aucMean	TRAP-6 (AUC) across concentration range	Optimul *	PRP ****	TRAP-6	1.5 µM – 1.8 mM	↑ platelet reactivity
TRAP6eMax	TRAP-6 maximal % aggregation (Optimul)	Optimul *	PRP ****	TRAP-6	1.5 µM – 1.8 mM	↑ platelet reactivity
TTAS_AUC	Platelet thrombus formation over collagen under shear (AUC)	TTAS ***	Whole blood ^	Collagen	Coated on surface	↑ platelet reactivity
U46619Agg20	U46619 concentration needed to reach 20% aggregation	Optimul *	PRP ****	U46619	0.005, 0.02, 0.10, 0.44, 1.98, 8.89, 40 µM	↓ platelet reactivity
U46619Agg40	U46619 concentration needed to reach 40% aggregation	Optimul *	PRP ****	U46619	0.005, 0.02, 0.10, 0.44, 1.98, 8.89, 40 µM	↓ platelet reactivity
U46619aucMean	U46619 (AUC) across concentration range	Optimul *	PRP ****	U46619	0.005, 0.02, 0.10, 0.44, 1.98, 8.89, 40 µM	↑ platelet reactivity
U46619ec50	U46619 concentration needed to reach 50% aggregation	Optimul *	PRP ****	U46619	0.005, 0.02, 0.10, 0.44, 1.98, 8.89, 40 µM	↓ platelet reactivity
U46619ecMax	U46619 concentration needed to reach maximal aggregation	Optimul *	PRP ****	U46619	0.005, 0.02, 0.10, 0.44, 1.98, 8.89, 40 µM	↓ platelet reactivity
U46619eMax	U46619 maximal % aggregation (Optimul)	Optimul *	PRP ****	U46619	0.005, 0.02, 0.10, 0.44, 1.98, 8.89, 40 µM	↑ platelet reactivity
U46619slope	U46619 slope across concentration range	Optimul *	PRP ****	U46619	0.005, 0.02, 0.10, 0.44, 1.98, 8.89, 40 µM	↑ platelet reactivity

Supplementary Table 1 Footnotes:

- * Optimul traits are derived from a plate-based concentration range
- ** Multiplate is a form of impedance aggregometry
- *** TTAS chips consist of collagen shear microchannels
- **** PRP derived from sodium citrated blood
- ^ Whole blood derived from hirudin anticoagulated blood

Supplementary Table 2: Significant associations that survived multiple testing with an FDR ≤ 0.05 for a model additionally adjusting for common medication use.

CpG	Annotated Gene (in or nearby)	Platelet Agonist	Platelet Assay	M1 Beta	M2 Beta	M1 Standard Error (SE)	M2 Standard Error (SE)	M1 P-value	M2 P-value	Multiple Test Adjusted P-value	M1 Rank	M2 Rank
cg24267699	*ABO*	Ristocetin	LTA	0.2139	0.2156	0.0314	0.0321	1.04E-11	2.02E-11	4.61E-06	1	1
cg21160290	*ABO*	Ristocetin	LTA	0.1792	0.1806	0.0280	0.0287	1.59E-10	3.37E-10	3.51E-05	2	2
cg22535403	*ABO*	Ristocetin	LTA	0.1713	0.1736	0.0278	0.0286	7.87E-10	1.28E-09	1.16E-04	3	3
cg11879188	*ABO*	Ristocetin	LTA	0.1702	0.1681	0.0280	0.0289	1.32E-09	6.06E-09	1.46E-04	4	5
cg25020897	*CAPRIN2*	ADP	Flow cytometry	0.1678	0.1641	0.0280	0.0280	2.17E-09	4.95E-09	9.64E-04	5	4
cg14285533	*LINC2848*	ADP	LTA	0.1178	0.1119	0.0202	0.0205	5.40E-09	4.73E-08	2.39E-03	6	13
cg22380533	*SYT5*	ADP	Flow Cytometry	0.1610	0.1595	0.0284	0.0284	1.51E-08	2.05E-08	3.35E-03	7	7
cg07762993	*KIF25-AS1*	Collagen	LTA	-0.1571	-0.1614	0.0281	0.0291	2.42E-08	3.13E-08	1.07E-02	8	10
cg10122766	*TLE6*	ADP	Flow cytometry	0.1607	0.1518	0.0292	0.0289	3.97E-08	1.55E-07	1.76E-02	9	20
cg10512202	*LIMD1*	Arachidonic acid	LTA	-0.1118	-0.1091	0.0204	0.0211	4.49E-08	2.42E-07	1.99E-02	10	24
cg25385322	*FIS1*	Ristocetin	LTA	-0.1618	-0.1433	0.0296	0.0304	4.67E-08	2.45E-06	2.07E-02	11	42
cg10122766	*TLE6*	ADP	Flow cytometry	0.1601	0.1507	0.0293	0.0290	4.75E-08	2.05E-07	2.11E-02	12	22
cg19372507	*MINAR1*	ADP	Optimul	0.1209	0.1204	0.0221	0.0221	4.83E-08	5.21E-08	2.14E-02	13	14
cg02936049	*ZBTB38*	ADP	LTA	0.1899	0.1776	0.0348	0.0347	4.83E-08	3.15E-07	2.14E-02	14	28
cg11772020	*LINC2537*	Arachidonic acid	Multiplate	-0.1997	-0.2004	0.0366	0.0366	5.20E-08	4.59E-08	2.30E-02	15	12
cg07762993	*KIF25-AS1*	Collagen	LTA	-0.1526	-0.1568	0.0280	0.0290	5.21E-08	6.68E-08	2.31E-02	16	15
cg07762993	*KIF25-AS1*	Collagen	LTA	-0.1526	-0.1568	0.0280	0.0290	5.21E-08	6.68E-08	2.31E-02	17	16
cg10512202	*LIMD1*	Arachidonic acid	LTA	-0.1105	-0.1065	0.0203	0.0210	5.27E-08	4.04E-07	2.34E-02	18	30
cg10512202	*LIMD1*	Arachidonic acid	LTA	-0.1105	-0.1065	0.0203	0.0210	5.27E-08	4.04E-08	2.34E-02	19	31
cg10512202	*LIMD1*	Arachidonic acid	LTA	-0.1094	-0.1055	0.0201	0.0208	5.52E-08	4.10E-07	2.44E-02	20	32
cg10321623	*B4GALNT3*	ADP	Optimul	0.1260	0.1249	0.0232	0.0232	5.76E-08	7.40E-08	1.28E-02	21	17
cg24965248	*FGF6*	Epinephrine	LTA	-0.1178	-0.1174	0.0217	0.0223	6.01E-08	1.56E-07	2.66E-02	22	21
cg22380533	*SYT5*	ADP	Flow cytometry	0.1583	0.1633	0.0294	0.0290	7.49E-08	1.95E-08	1.66E-02	23	6
cg04738774	*CALD1*	ADP	Optimul	-0.1608	-0.1628	0.0299	0.0293	7.74E-08	3.01E-08	3.43E-02	24	9

cg25385322	*FIS1*	Ristocetin	LTA	-0.1575	-0.1373	0.0294	0.0304	8.80E-08	5.54E-06	3.90E-02	25	44
cg26962595	*STARD10*	None	Flow cytometry	-0.1511	-0.1545	0.0284	0.0291	1.10E-07	1.19E-07	4.87E-02	26	19
cg20732755	*PRKN*	ADP	LTA	-0.1034	-0.0953	0.0197	0.0201	1.56E-07	2.15E-06	3.45E-02	27	40
cg01078871	*FBXO33*	ADP	Flow cytometry	0.1463	0.1495	0.0280	0.0280	1.81E-07	9.82E-08	2.68E-02	28	18
cg23201265	*LINC2955*	Ristocetin	LTA	0.1465	0.1473	0.0281	0.0289	1.93E-07	3.54E-07	4.27E-02	29	29
cg25385322	*FIS1*	Arachidonic acid	LTA	-0.1097	-0.0874	0.0210	0.0218	1.93E-07	6.02E-05	4.29E-02	30	46
cg19342368	*WHAMM*	ADP	Optimul	0.1532	0.1581	0.0295	0.0289	2.12E-07	4.57E-08	4.70E-02	31	11
cg20417128	*PXDN*	Ristocetin	LTA	0.1471	0.1402	0.0283	0.0294	2.19E-07	1.86E-06	4.84E-02	32	38
cg20417128	*PXDN*	Ristocetin	LTA	0.1472	0.1398	0.0284	0.0294	2.25E-07	2.04E-06	3.32E-02	33	39
cg06213060	*LINC00690*	ADP	Optimul	-0.1526	-0.1527	0.0297	0.0297	3.00E-07	2.86E-07	4.44E-02	34	27
cg25287211	*CLN5*	ADP	Optimul	-0.1144	-0.1153	0.0223	0.0223	3.22E-07	2.58E-07	3.56E-02	35	26
cg01294058	*CDKN1C*	ADP	LTA	-0.0973	-0.0961	0.0191	0.0194	3.70E-07	8.13E-07	4.10E-02	36	36
cg03892812	*KNDC1*	ADP	Optimul	0.1242	0.1271	0.0245	0.0245	4.06E-07	2.10E-07	3.60E-02	37	23
cg20206224	*XPO5*	ADP	Flow cytometry	0.1414	0.1318	0.0279	0.0279	4.15E-07	2.37E-06	4.60E-02	38	41
cg14757344	*RRP1*	ADP	Flow cytometry	0.1433	0.1459	0.0283	0.0283	4.27E-07	2.57E-07	3.78E-02	39	25
cg18905668	*GTF2H1*	ADP	LTA	0.1243	0.1008	0.0246	0.0249	4.34E-07	5.25E-05	3.85E-02	40	45
cg04970158	*PLCD3*	ADP	LTA	0.1007	0.0947	0.0200	0.0203	4.91E-07	3.06E-06	3.63E-02	41	43
cg04231958	*UBE3B*	ADP	Flow cytometry	0.1445	0.1626	0.0290	0.0290	6.50E-07	2.14E-08	4.80E-02	42	8
cg00814244	*MYCN*	ADP	Optimul	-0.1076	-0.1069	0.0216	0.0216	6.51E-07	7.75E-07	4.81E-02	43	34
cg14823535	*SUN1*	ADP	Optimul	0.1050	0.1050	0.0213	0.0212	8.10E-07	8.05E-07	4.49E-02	44	35
cg09249084	*WLS; GNG12-AS2*	ADP	Optimul	0.1057	0.1034	0.0214	0.0214	8.58E-07	1.44E-06	4.23E-02	45	37
cg10948783	*FAM241A*	ADP	Optimul	-0.1081	-0.1099	0.0221	0.0221	1.01E-06	6.54E-07	4.47E-02	46	33

Supplementary Table 2 Footnotes: M1 denotes the model unadjusted for common medications (except aspirin at Exam 3) and M2 denotes the model adjusted for P2Y12 inhibitors, and antiplatelet, antidepressant, and anticoagulant drugs at Exam 3. Rank denotes the lowest P-values for each model ranked smallest to largest.

Supplementary Table 3: Megakaryocyte methylation overlaps in Blueprint

CpG ID	Annotated Gene	CHR	Location in Genome (hg38)	Total Overlaps in Blueprint	Size of Region (bp)	Number of CpGs in Region	S004AV Call Type	S004AV Average Methylation Level	S004BT Call Type	S004BT Average Methylation Level
cg26962595	STARD10	11	72,793,844	10	515	49			hypo	0.00257
					503	48	hypo	0.00023		
cg25385322	FIS1	7	101,240,813	8	3,374	73			hyper	0.91536
					1,854	58	hyper	0.93903		
cg24267699	ABO	9	133,275,943	8	1,284	139	hypo	0.01860		
					1,075	130			hypo	0.00965
cg11879188	ABO	9	133,274,492	8	2,279	36	hyper	0.84533		
					1,700	38			hyper	0.87918
cg10122766	TLE6	19	2,977,388	8	696	32			hypo	0.00134
					664	31	hypo	0.00390		
cg14757344	RRP1	21	43,789,626	8	1,250	88	hypo	0.01020		
					1,159	67			hypo	0.01104
cg22380533	SYT5	19	55,179,373	8	1,803	55	hypo	0.02025		
					1,038	41			hypo	0.00224
cg22535403	ABO	9	133,274,616	7	2,279	36	hyper	0.84533		
					1,700	38			hyper	0.87918
cg21160290	ABO	9	133,274, 525	7	2, 279	36	hyper	0.84533		
					1,700	38			hyper	0.87918
cg01294058	CDKN1C	11	2,884,324	7	2,638	281			hypo	0.01696
					2,731	269	hypo	0.01838		
cg25287211	CLN5	13	76,992,787	7	1,217	67	hypo	0.01299		
					1,072	71			hypo	0.01128
cg01078871	FBXO33	14	39,432,304	7	1,719	100	hypo	0.02025		
					934	83			hypo	0.00224
cg10512202	LIMD1	3	45,607,801	7	11,884	156	hyper	0.94652	hyper	0.94755
cg04231958	UBE3B	12	109,477,346	6						
cg18905668	GTF2H1	11	18,366,600	6	2,168	21			hyper	0.95200
cg00814244	MYCN	2	15,939,565	6	3,681	13			hyper	0.91200
					2,264	191	hypo	0.00073		
cg20206224	XPO5	6	43,576,034	6	630	59	hypo	0.00159		

					595	45			hypo	0.00089
cg10321623	B4GALNT3	12	561,354	6	7,861	156	hyper	0.93812		
					5,108	119			hyper	0.94147
cg10948783	FAM241A	4	112,146,209	5						
cg25020897	CAPRIN2	12	30,754,662	5						
cg11772020	LINC2537	6	43,838,733	5	13,932	118	hyper	0.92141		
					8,315	73			hyper	0.93519
cg14285533	LINC2848	7	63,925,950	5	148	10			hypo	0.11170
					983	11	hypo	0.05782		
cg14823535	SUN1	7	843,922	5	55,213	1,255			hyper	0.95663
					40,808	1,052	hyper	0.95013		
cg19342368	WHAMM	15	82,809,503	4						
cg20417128	PXDN	2	1,686,853	3	10,874	202	hyper	0.91111		
					5,600	120			hyper	0.92885
cg07762993	KIF25-AS1	6	167,990,884	3	17,155	453			hyper	0.91455
					6,722	211	hyper	0.87555		
cg04738774	CALD1	7	134,788,470	3	1,801	6			hyper	0.93517
					7,310	52	hyper	0.95635		
cg04970158	PLCD3	17	45,131,373	3	10,108	150	hyper	0.92617		
					10,166	135			hyper	0.92799
cg09249084	WLS; GNG12-AS2	1	68,194,211	2	6,552	35			hyper	0.90966
					7,184	41	hyper	0.87678		
cg20732755	PRKN	6	161,727,121	2	17,787	125	hyper	0.83545		
					3,029	18			hyper	0.92822
cg03892812	KNDC1	10	133,207,427	2	10,360	414			hyper	0.89432
					9,764	405	hyper	0.88391		
cg24965248	FGF6	12	4,445,774	2	17,504	179	hyper	0.87154		
					15,473	156			hyper	0.89950
cg23201265	LINC2955	12	22,925,302	2	6,948	26	hyper	0.85677		
					1,371	3			hyper	0.86800
cg02936049	ZBTB38	3	141,383,757	1						
cg06213060	LINC00690	3	16,536,219	1						
cg19372507	MINAR1	15	79,429,686	1						

Supplementary Table 4: Megakaryocyte DNAse activity overlaps in Blueprint.

CpG ID	Annotated Gene	CHR	Location in Genome (hg38)	Total Overlaps in Blueprint	C006NS *(exp. 1)* DNAse Peak Intensity	Size of Region (bp)	C006NS *(exp. 2)* DNAse Peak Intensity	Size of Region (bp)	S004BT DNAse Peak Intensity	Size of Region (bp)
cg26962595	STARD10	11	72,793,844	10			29.297	150	36.922	150
cg25385322	FIS1	7	101,240,813	8						
cg24267699	ABO	9	133,275,943	8					7.578	150
cg11879188	ABO	9	133,274,492	8						
cg10122766	TLE6	19	2,977,388	8	27.875	150	16.328	150	16.266	150
cg14757344	RRP1	21	43,789,626	8			16.563	150	19.656	150
cg22380533	SYT5	19	55,179,373	8	14.688	150				
cg22535403	ABO	9	133,274,616	7						
cg21160290	ABO	9	133,274,525	7						
cg01294058	CDKN1C	11	2,884,324	7						
cg25287211	CLN5	13	76,992,787	7					9.953	150
cg01078871	FBXO33	14	39,432,304	7			29.922	150		
cg10512202	LIMD1	3	45,607,801	7						
cg04231958	UBE3B	12	109,477,346	6	26.906	150	19.094	150	18.922	150
cg18905668	GTF2H1	11	18,366,600	6						
cg00814244	MYCN	2	15,939,565	6					8.094	150
cg20206224	XPO5	6	43,576,034	6			23.250	150	20.484	150
cg10321623	B4GALNT3	12	561,354	6						
cg10948783	FAM241A	4	112,146,209	5					8.521	150
cg25020897	CAPRIN2	12	30,754,662	5			7.391	150		
cg11772020	LINC2537	6	43,838,733	5						
cg14295533	LINC2848	7	63,925,950	5						
cg14823535	SUN1	7	843,922	5						
cg19342368	WHAMM	15	82,809,503	4	46.906	150	23.484	150	26.016	150
cg20417128	PXDN	2	1,686,853	3						
cg07762993	KIF25-AS1	6	167,990,884	3						
cg04738774	CALD1	7	134,788,470	3						
cg04970158	PLCD3	17	45,131,373	3						
cg09249084	WLS; GNG12-AS2	1	68,194,211	2						

cg20732755	PRKN	6	161,727,121	2	
cg03892812	KNDC1	10	133,207,427	2	
cg24965248	FGF6	12	4,445,774	2	
cg23201265	LINC2955	12	22,925,302	2	
cg02936049	ZBTB38	3	141,383,757	1	
cg06213060	LINC00690	3	16,536,219	1	
cg19372507	MINAR1	15	79,429,686	1	

Supplementary Table 5: Megakaryocytes histone modification overlaps in Blueprint.

Cpg ID	Annotated Gene	CHR	Location in Genome (hg38)	Total Overlaps in Blueprint	S004BT				S00VHK						S004AV		
					H3K4me1	H3K36me3	H3K4me3	H3K27ac	H3K4me1	H3K27ac	H3K9me3	H3K36me3	H3K27me3	H3K4me3	H3K4me1	H3K4me3	H3K36me3
cg26962595	STARD10	11	72,793,844	10	42<br>*3,195*				66<br>*15,240*	431<br>*1,064*				3,165<br>*2,030*	75<br>*4,212*	231<br>*542*	
cg25385322	FIS1	7	101,240,813	8	44<br>*3,616*	20<br>*1,249*			89<br>*8,890*			32<br>*13,189*			69<br>*4,852*		25<br>*9,326*
cg24267699	ABO	9	133,275,943	8				32<br>*205*	52<br>*9,679*				34<br>*6,705*	1,884<br>*2,230*	17<br>*287*		
cg11879188	ABO	9	133,274,492	8	50<br>*7,361*				52<br>*9,679*	68<br>*223*			34<br>*6,705*	1,884<br>*2,230*	35<br>*7,407*		
cg10122766	TLE6	19	2,977,388	8					80<br>*1,520*					628<br>*810*	42<br>*1,518*		
cg14757344	RRP1	21	43,789,626	8			42<br>*180*			590<br>*525*				4,542<br>*2,435*		194<br>*679*	
cg22380533	SYT5	19	55,179,373	8	23<br>*1,567*				89<br>*2,121*				46<br>*15,536*	155<br>*1,046*	33<br>*1,723*		
cg22535403	ABO	9	133,274,616	7	50<br>*7,361*				52<br>*9,679*	68<br>*223*			34<br>*6,705*	1,884<br>*2,230*			
cg21160290	ABO	9	133,274,525	7	50<br>*7,361*				52<br>*9,679*	68<br>*223*			34<br>*6,705*	1,884<br>*2,230*			
cg01294058	CDKN1C	11	2,884,324	7	23<br>*1,578*				42<br>*6,925*				47<br>*2,058*	1,791<br>*2,988*	38<br>*2,897*		
cg25287211	CLN5	13	76,992,787	7				63<br>*428*		663<br>*955*				3,960<br>*2,173*		292<br>*874*	
cg01078871	FBXO33	14	39,432,304	7				749<br>*435*		1,641<br>*1,601*				4,840<br>*4,241*		293<br>*1,847*	
cg10512202	LIMD1	3	45,607,801	7	35<br>*5,311*				59<br>*11,061*			44<br>*16,967*			36<br>*7,849*		32<br>*13,563*
cg04231958	UBE3B	12	109,477,346	6						1,377<br>*1,984*				4,050<br>*2,957*		221<br>*1,685*	

cg18905668	GTF2H1	11	18,366,600	6	51<br>*4,171*				101<br>*8,862*			77<br>*44,816*		170<br>*749*	66<br>*4,573*		
cg00814244	MYCN	2	15,939,565	6					35<br>*7,826*					3,447<br>*6,216*	30<br>*2,445*		
cg20206224	XPO5	6	43,576,034	6										4,780<br>*3,393*		303<br>*1,026*	
cg10321623	B4GALNT3	12	561,354	6	47<br>*3,195*				96<br>*7,453*	370<br>*2,740*					64<br>*7,796*		
cg10948783	FAM241A	4	112,146,209	5						275<br>*787*				4,075<br>*2,788*	40<br>*2,520*	183<br>*996*	
cg25020897	CAPRIN2	12	30,754,662	5			56<br>*434*			108<br>*354*				4,202<br>*2,474*		162<br>*746*	
cg11772020	LINC2537	6	43,838,733	5	27<br>*1,028*				75<br>*4,183*						65<br>*2,344*		
cg14295533	LINC2848	7	63,925,950	5						199<br>*552*				1,679<br>*2,393*		51<br>*654*	
cg14823535	SUN1	7	843,922	5		34<br>*3,067*						64<br>*43,186*					31<br>*8,342*
cg19342368	WHAMM	15	82,809,503	4										4,373<br>*3,292*			
cg20417128	PXDN	2	1,686,853	3								44<br>*24,683*					
cg07762993	KIF25-AS1	6	167,990,884	3							24<br>*1,295*						
cg04738774	CALD1	7	134,788,470	3											55<br>*24,107*		
cg04970158	PLCD3	17	45,131,373	3					39<br>*2,809*								
cg09249084	WLS; GNG12-AS2	1	68,194,211	2													
cg20732755	PRKN	6	161,727,121	2													
cg03892812	KNDC1	10	133,207,427	2													
cg24965248	FGF6	12	4,445,774	2													
cg23201265	LINC2955	12	22,925,302	2													
cg02936049	ZBTB38	3	141,383,757	1					19								

									545								
cg06213060	LINC00690	3	16,536,219	1					18<br>*1,527*								
cg19372507	MINAR1	15	79,429,686	1									81<br>*9,415*				

Supplementary Table 4 Footnotes:

- Cells with overlapping ChIP-seq activity are split horizontally, where the top, unitalicized value is the score for that particular modification, and the italicized value underneath it is the size of the assayed region in base pairs (bp).

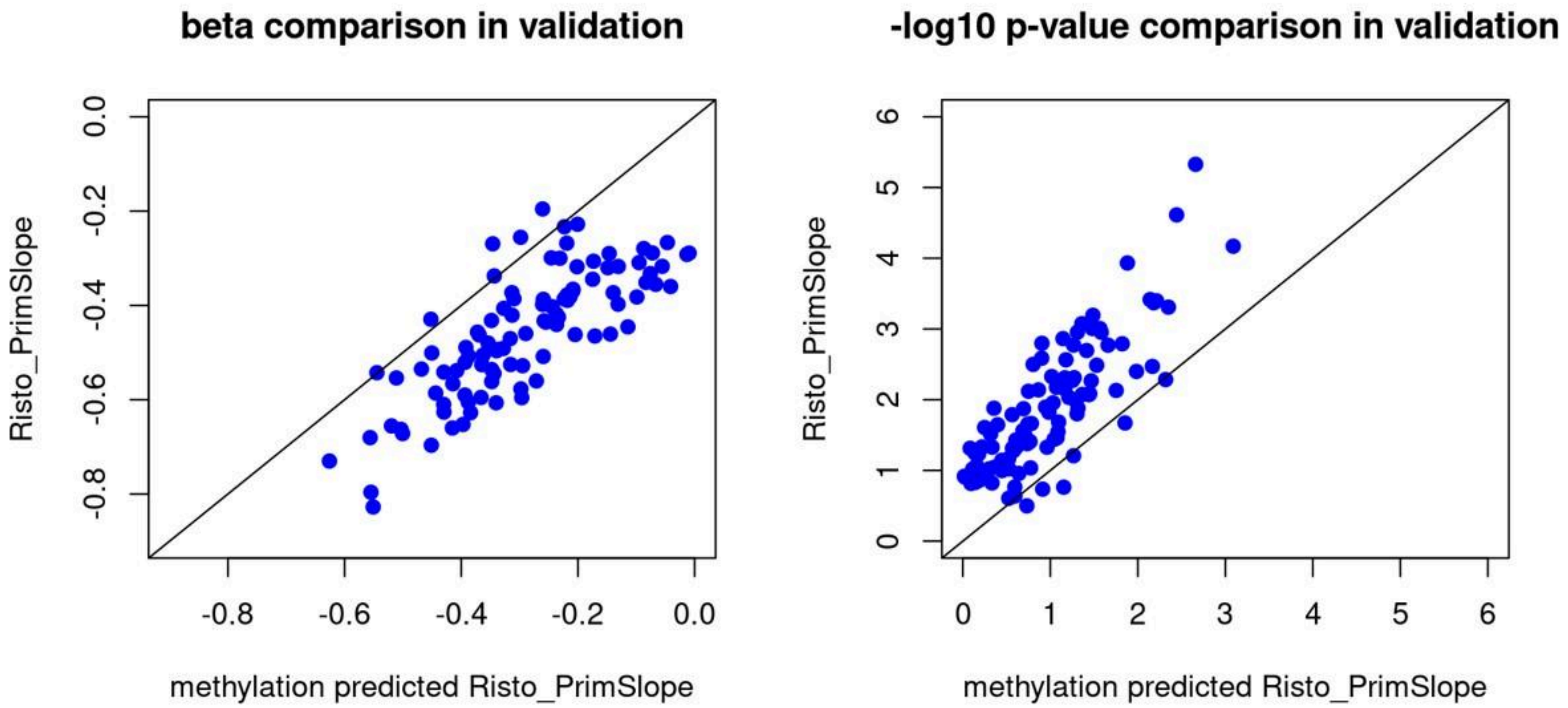

Supplemental Figure 1: Scatter plots of peak VO2 estimates and -log10 *P*-values from the two association tests (Risto_PrimSlope and the predictor) for validation.

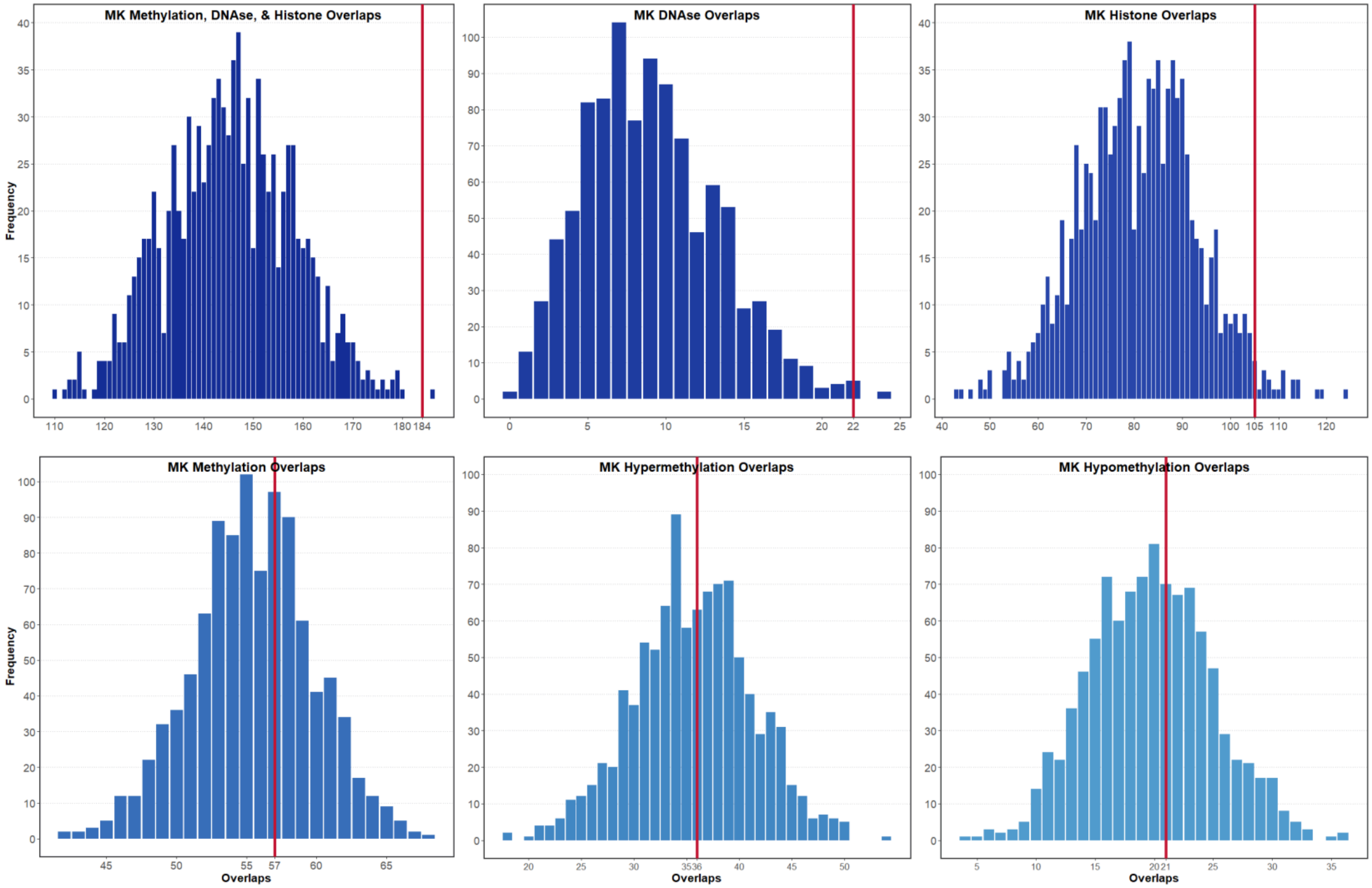

Supplemental Figure 2: Distribution of number of overlaps for MK methylation, DNAse, & ChIP-seq (histones); MK DNAse; MK ChIP-seq (histones); MK methylation; MK hypermethylation; and MK hypomethylation. The number of overlaps in our subset is highlighted by a break in the x axis and a vertical red line.

Supplementary Method 1: DNA methylation-based predictor using Elastic Net.

The selected trait of interest was Risto_PrimSlope, a measure of the primary wave slope of ristocetin responses in the LTA platform. In addition to 4 signals in *ABO* in our study, there is also a known association of Risto_PrimSlope with peak oxygen intake (VO2) reported in Table 2 of a previous work.[1] 100 replications were used to evaluate the methylation-predicted Risto_PrimSlope. For each replication, the same procedure described below was utilized.

The 1,286 samples in our Risto_PrimSlope epigenome-wide association study (EWAS) were split into training (N = 800) and validation (N = 486) datasets. From the Risto_PrimSlope EWAS, 4,406 CpGs without missing values and with a P-value of < 0.01 were selected and standardized for elastic net analysis. Normalization (feature scaling) was applied to beta and -log10 P-values of the 4,406 CpGs. Both these values were combined with an equal weight of 0.5 to construct a hybrid weight for an adaptive penalty. This was done in order to apply less shrinkage to CpGs with stronger evidence.

Then the Risto_PrimSlope data, standardized CpGs, and the adaptive penalty were used in a 10-fold cross-validation elastic net analysis using the *cv.glmnet* function in the *glmnet* R package[2] with the mixing parameter alpha set to be 0, 0.25, 0.5, 0.75 and 1 for selecting best results with the minimum mean squared error. The alpha and lambda1.se from the best results were then provided (in addition to Risto_PrimSlope data, standardized CpGs, and the adaptive penalty) to fit *glmnet.* This was performed in order to extract coefficients and weights for computing the predictor using methylation data in the validation dataset.

Supplementary Method 2: Platelet mass spectrometry method.

Sample collection: Healthy controls above 18 years of age were enrolled in National Institutes of Health biosampling studies (ClinicalTrials.gov Identifier: NCT05403151). The participants consisted of 15 men and 15 women, with an average age of 31.06. None of the healthy controls were on immunomodulators, antiplatelet or anticoagulant medications. All protocols were reviewed and approved by the NIH Institutional Review Board and study participants were provided with written informed consent prior to participation, per the ICH E6 Guidelines for Good Clinical Practice originating from the Declaration of Helsinki.

Sample preparation: Platelet pellets were resuspended in 200 uL EasyPep^{TM} (Thermo Scientific) Buffer with 1 uL nuclease, protease inhibitors and Phos Stop. BCA was used to estimate protein concentration and 110 ug of each sample was combined with 50 uL Reduction and 50 uL alkylation solution. 4 ug of Trypsin/LysC was added to each sample and allowed to digest shaking at 750rpm overnight at 37°C. A pool sample was created by combining 10 ug of tryptic peptides from each sample and using 100 ug total for labeling The next day 200 ug TMT label was added to each sample and allowed to label for 1h at 25°C. Reactions were quenched with 50 uL of 5% hydroxylamine, 20% Formic acid for 10 minutes and samples were combined and cleaned up using the EasyPepTM (Thermo Scientific) Maxi Kit. Eluted samples were dried in the speed-vac.

Phosphopeptide Enrichment: The dried peptide mixture was enriched for phosphorylation site containing peptides sequentially using the High Select titanium dioxide kit (Thermo) followed by the Fe-NTA kit (IMAC, Thermo) according to the procedure in the product manuals. The peptides were first enriched by TiO2, and the flow-through and wash was saved, dried, and enriched by IMAC. The eluted phosphopeptides from each enrichment were combined and dried for MS analysis. The IMAC flow-through and wash was dried and saved for high-pH reverse fractionation.

High pH Fractionation of IMAC-FT: The IMAC-FT peptides were resuspended in 100 uL of LC/MS grade water and 85uL was fractionated by High-pH reverse phase liquid chromatography using a Waters Acquity UPLC system coupled with a fluorescence detector (Waters, Milford, MA). Separation was performed on a 150mm x 3.0mm Xbridge Peptide BEMTM 2. 5 mm C18 column (Waters, MA) operating at 0.35 ml/min. The column was washed with mobile phase A (10 mM Ammonium Formate, pH 9.4) for 5 min followed by gradient elution 10- 50% B (10 mM Ammonium Formate/90% Acetonitrile pH 9.6, 5-60 min) and 50-75 %B (60-70 min). The fractions were collected every minute. These 60 fractions were pooled into 12 fractions. The fractions were vacuum centrifuged to dryness and stored at -80oC until analysis by mass spectrometry.

LC/MS/MS analysis: Each fraction was resuspended in 50 uL of 0.1% FA and 3 uL was loaded onto a Dionex U3000 RSLC attached to an Orbitrap Eclipse (Thermo) equipped with a FAIMS and EasySpray ion source. Solvent A was comprised of 0.1% FA and Solvent B was 0.1% FA in 80% CAN. The gradient pump was run at 300 uL/min with an LC gradient of 5-10%B for 5 min, 7-

35%B for 85 min, 35-50%B for 25 min, 50-95%B for 4min, 95%B for 7 min then a re-equilibration of the column at 5%B for 17 min. The MS was run in the TopSpeed method with three FAIMS compensation voltages (-50, -65, -80). Spray voltage was set at 2200V and the ion transfer tube was at 300°C. MS1 scans were acquired in the Orbitrap at 120,000 resolution, AGC of 4e5, and max injection time of 50ms in a mass range of 375-1600 m/z. MS2 scans were acquired in the Orbitrap using the TurboTMT method with a resolution of 15,000, intensity threshold 2.5e4, AGC 5e4, HCD energy 30%, isolation window 1.6 m/z and charges of 2-5 for MS2 selection. Monoisptopic Precursor selection (MIPs), Easy-IC for internal calibration and advanced peak determination were enabled.

For phosphopeptide analysis the gradient pump was run at 300 uL/min with an LC gradient of 5-10%B for 5 min, 10-12% for 1 minute, 12-35%B for 134 min, 35-50%B for 35 min, 50-95%B for 4min, 95%B for 7 min then a re-equilibration of the column at 5%B for 17 min. Two FAIMS methods were used with the cycle time of each FAIMS CV being 0.75 seconds (3 seconds total): Method #1 CVs = -45, -55, -65, -75; Method #2 CVs = -50, -60 -70, -80.

Database search: The MS files were searched in Proteome discoverer using the Sequest node. Data was searched against the Uniprot human data base using full trypsin digestion with a max 2 missed cleavages, Min peptide length 6, MS1 mass tolerance of 10ppm, MS2 tolerance of 0.02 Da, variable modification of oxidation on methionine and static modifications of carbamidomethyl on cysteine, phosphorylation on serine, threonine, and tyrosine, and TMTpro on lysine and peptide N-terminus. Percolator was used for FDR analysis and TMTPro reporter ions were quantified using the Reporter Ion Quantifier node and normalized on total peptide intensity on each channel.

References:

1. GRECH, J., et al., *Cardiorespiratory Fitness Is Associated with Decreased Platelet Reactivity.* Medicine & Science in Sports & Exercise, 2024. **56**(11): p. 2195–2202.
2. Tay, J.K., B. Narasimhan, and T. Hastie, *Elastic Net Regularization Paths for All Generalized Linear Models.* Journal of statistical software, 2023. **106**: p. 1.
